# Japan Society of Clinical Oncology provisional clinical opinion for the diagnosis and use of immunotherapy in patients with deficient DNA mismatch repair tumors, cooperated by Japanese Society of Medical Oncology, First Edition

**DOI:** 10.1007/s10147-019-01498-8

**Published:** 2019-07-08

**Authors:** Saori Mishima, Hiroya Taniguchi, Kiwamu Akagi, Eishi Baba, Yutaka Fujiwara, Akira Hirasawa, Masafumi Ikeda, Osamu Maeda, Kei Muro, Hiroshi Nishihara, Hiroyki Nishiyama, Tadao Takano, Katsuya Tsuchihara, Yasushi Yatabe, Yasuhiro Kodera, Takayuki Yoshino

**Affiliations:** 1 Department of Gastrointestinal Oncology, National Cancer Center Hospital East, 6-5-1 Kashiwanoha, Kashiwa, Chiba 277-8577 Japan; 2 Division of Molecular Diagnosis and Cancer Prevention, Saitama Cancer Center, Saitama, Japan; 3 Department of Oncology and Social Medicine, Graduate School of Medical Sciences, Kyushu University, Fukuoka, Japan; 4 Department of Respiratory Medicine, Mitsui Memorial Hospital, Tokyo, Japan; 5 Department of Clinical Genomic Medicine, Graduate School of Medicine, Dentistry and Pharmaceutical Sciences, Okayama University, Okayama, Japan; 6 Department of Hepatobiliary and Pancreatic Oncology, National Cancer Center Hospital East, Kashiwa, Japan; 7 Department of Clinical Oncology and Chemotherapy, Nagoya University Hospital, Nagoya, Japan; 8 Department of Clinical Oncology, Aichi Cancer Center Hospital, Nagoya, Japan; 9 Genomics Unit, Keio Cancer Center, Keio University, Tokyo, Japan; 10 Department of Urology, Tsukuba University, Tsukuba, Japan; 11 Department of Gynecology and Obstetrics, Tohoku University, Sendai, Japan; 12 Department of Translational Research, Exploratory Oncology Research and Clinical Trial Center, National Cancer Center, Tokyo, Japan; 13 Department of Pathology and Molecular Diagnostics, Aichi Cancer Center Hospital, Nagoya, Japan; 14 Department of Gastrointestinal Surgery, Nagoya University, Nagoya, Japan

**Keywords:** Mismatch repair-deficient advanced solid tumor, dMMR, MSI-H, PD-1/PD-L1 inhibitor, Provisional clinical opinion

## Abstract

**Background:**

Novel therapeutic agents have improved survival outcomes in patients with advanced solid tumors. In parallel, the development of predictive biomarkers to identify patients who are likely to benefit from a certain treatment has also contributed to the improvement of survival. Recently, clinical trials have reported the efficacy of immune checkpoint inhibitors in the treatment of mismatch repair-deficient (dMMR) advanced solid tumors. In Japan, a PD-1 inhibitor for dMMR advanced solid tumors, regardless of the primary tumor site, has been approved. However, there are some issues related to administering immune checkpoint inhibitors in the clinical practice setting, making it necessary to develop the guidelines.

**Methods:**

Clinical questions (CQs) regarding medical care were formulated for patients with dMMR advanced solid tumors, and evidence to the CQs was collected by manual search to prepare recommendations. Then, the committee members voted to determine the level of each recommendation considering the strength of evidence, expected risks and benefits to patients, and other factors.

**Results:**

The current guideline, which we consider a provisional clinical opinion at this point, describes the 11 requirements to be considered in terms of patients for whom dMMR testing is recommended, the timing and methods of dMMR testing, and clinical care systems required to perform dMMR testing properly and to administer immune checkpoint inhibitors safely.

**Conclusion:**

This provisional clinical opinion proposes the requirements for performing dMMR testing properly to select patients who are likely to benefit from immune checkpoint inhibitors and administering them safely.

**Electronic supplementary material:**

The online version of this article (10.1007/s10147-019-01498-8) contains supplementary material, which is available to authorized users.

## Summary

In recent years, many clinical trials have reported the efficacy of immune checkpoint inhibitors in the treatment of advanced solid tumors with deficient DNA mismatch repair (dMMR). In Japan, PD-1 inhibitor for advanced/recurrent microsatellite instability-high (MSI-H) solid tumors, regardless of the primary tumor site, has been approved. This has made it necessary to develop reference manuals, including guidelines, which enable smooth implementation of testing and treatment in the clinical setting.

This provisional clinical opinion proposes the following 11 requirements regarding the dMMR testing performed to select patients who are likely to benefit from PD-1/PD-L1 inhibitors.

1. For patients with solid tumors who are receiving standard systemic treatment or who have difficulty receiving any standard treatment, dMMR testing is highly recommended to determine eligibility for PD-1/PD-L1 inhibitors.
2. For patients with unresectable solid tumors, irrespective of MMR status, for which clinical application of PD-1/PD-L1 inhibitors has already been approved, dMMR testing should be considered to determine eligibility for PD-1/PD-L1 inhibitors.
3. For patients with solid tumors that are curable with local treatment, dMMR testing for determining eligibility for PD-1/PD-L1 inhibitors is not recommended.
4. For patients with solid tumors who have already undergone treatment with PD-1/PD-L1 inhibitors, dMMR testing for redetermining eligibility for PD-1/PD-L1 inhibitors is not recommended.
5. When a tumor is detected in patients already diagnosed with Lynch syndrome, dMMR testing for determining eligibility for PD-1/PD-L1 inhibitors is recommended.
6. As dMMR testing for determining eligibility PD-1/PD-L1 inhibitors, microsatellite instability (MSI) testing is highly recommended.
7. As dMMR testing for determining eligibility for PD-1/PD-L1 inhibitors, immunohistochemistry (IHC) is recommended.
8. As dMMR testing for determining eligibility for PD-1/PD-L1 inhibitors, an NGS testing approach for which analytical validity has been established is recommended.
9. It is highly recommended to carry out dMMR testing in an environment that can ensure technical accuracy and the quality of the results.
10. It is highly recommended to carry out dMMR testing in an environment with established genetic diagnostic and genetic counseling systems.
11. It is highly recommended that immune checkpoint inhibitors are used in an environment, where adequate measures can be taken in response to immune-related adverse events.

In Europe and the US, MSI testing and mismatch repair protein immunostaining are the most common dMMR-testing methods. However, these testing methods are expected to shift to next-generation sequencing (NGS) in the near future. Please keep in mind that this provisional clinical opinion, which also includes such future trends, will be revised in a timely manner, along with continuously and steadily advancing cancer treatment and new knowledge on biomarkers, including dMMR.

## About the guidelines

### The necessity and purposes of the guidelines

In Japan, approximately 380,000 people die of malignant neoplasm (cancer) annually, and cancer is the number one cause of death. Improving the outcome of cancer treatment is a critical issue for the Japanese public. In the field of cancer pharmacotherapy, the advent of effective novel therapeutic drugs has improved treatment outcomes and prognoses. In parallel, the development of biomarkers to identify patients for whom a certain treatment is expected to be effective before starting treatment has contributed to the improvement of cancer treatment outcomes.

In December 2018, in Japan, pembrolizumab, a PD-1 inhibitor, was approved for advanced/recurrent MSI-H solid tumors. This is the first drug in Japan for tumor-agnostic indications. This treatment is expected to be a novel treatment option for solid tumors that are difficult to cure, while there are some issues related to administering the treatment in the clinical setting:

1. Because many clinical departments of different specialties are involved in diagnosis and treatment, different medical cares may be performed depending on the clinical department or the organ affected by cancer, causing confusion at clinical sites.
2. Tests that are used to judge the applicability of treatment, such as microsatellite instability testing, have a low degree of recognition.
3. Adverse events specific to immune checkpoint inhibitors need to be handled.
4. Because tests for this treatment lead to screening for Lynch syndrome, a system for genetic diagnosis, and treatment needs to be established.

For the issues described above, the various clinical practice guidelines published to date only briefly describe key points in the use of immune checkpoint inhibitors in patients with dMMR solid tumors. Since no comprehensive guidelines cover all key points regardless of primary tumor site, it is important to integrate common, tumor-agnostic views to the extent possible and provide a guide for clinical care to prevent confusion at clinical sites.

The current guidelines systematically describe items to be considered when seeing patients with dMMR solid tumors, including the timing and methods of testing defective mismatch repair function, the positioning of PD-1/PD-L1 inhibitor therapy, and clinical care systems. Moreover, given that recent progress in analytical techniques is facilitating rapid development of comprehensive genetic testing methods using next-generation sequencing and somatic cell genetic testing methods using blood samples (liquid biopsy), these novel testing methods are also included. In the clinical setting in Japan, if appropriate tests are performed on appropriate patients and the patients receive appropriate treatment at appropriate timing based on the recommended levels described in the present guidelines, treatment outcomes in patients with solid tumors are expected to be improved.

### Determination of recommended levels

In the preparation of the guidelines, clinical questions (CQ) were formulated, and evidence for answers to the CQs was gathered by handsearch. Based on the search results, the committee members voted to determine a recommended level for each CQ (Table 1). The recommended levels were determined by taking into account the strength of evidence for each CQ, expected benefits and losses of patients, and other factors. In voting, whether the contents of medical care (including tests and indications) are approved or covered by health insurance in Japan was not considered. However, relevant information was described in the remarks column as needed. The committee’s opinions were determined in the following manner: (1) if SR accounted for at least 70% of the vote, the committee’s opinion was SR; (2) if (1) was not met, but SR + R accounted for at least 70% of the vote, the committee’s opinion was R; (3) if (1) or (2) was not met, but SR + R + ECO accounted for at least 70% of the vote, the committee’s opinion was ECO; (4) if NR accounted for at least 50% of the vote, the committee’s opinion was NR, irrespective of the results of (1)–(3); and (5) if none of (1)–(4) was met, there was “no recommended level.”

**Table 1:** Degrees of recommendation and decision criteria

Degree of recommendation	Decision criteria
Strong recommendation (SR)	There is sufficient evidence and the benefits of testing outweigh the losses for patients
Recommendation (R)	There is certain evidence, considering the balance between benefits and losses for patients
Expert consensus opinion (ECO)	A certain consensus has been obtained although evidence and information that shows patient benefits cannot be said to be sufficient
No recommendation (NR)	There is no evidence

At present, some recommendations for CQs are not based on sufficient evidence. It is also possible that the accumulation of new evidence in the future will lead to substantial changes in the descriptions in the text and recommended levels. Consequently, the guidelines are positioned as a “provisional clinical opinion,” taking into account that the guidelines contain many recommendations made based on a consensus among the committee members at the current level.

## Introduction

### Cancer and mismatch repair function

Repairing non-complementary base pairs (mismatch) that are produced during DNA replication (mismatch repair: MMR) is an essential function for maintaining genome homeostasis. The condition where the MMR function is reduced is described as MMR deficient (dMMR) and the condition where the MMR function is maintained is described as MMR proficient (pMMR). Methods of evaluating the loss of MMR function include MSI testing, the immunohistochemistry (IHC) of MMR proteins, and NGS (refer to “dMMR testing methods” for details). The reduced MMR function changes the number of repeats of one-base to several-base repeat sequences (microsatellites). This phenomenon is called microsatellite instability. Microsatellite instability is considered to lead to accumulated mutations due to abnormal repairs in gene groups involved in tumor suppression, cell proliferation, DNA repair, apoptosis, etc., and thus contribute to the development and growth of tumors. The condition where microsatellite instability is detected with a high frequency is described as MSI-high (MSI-H) and the condition where microsatellite instability is detected with a low frequency or not detected is described as MSI low/microsatellite stable (MSI-L/MSS).

In some cancers, a reduced MMR function is detected. The reduced MMR function is mainly caused by MMR gene mutations and decreased expression of MMR genes due to abnormal methylation of the promoter region. A condition in which pathogenic variants of the *MLH1*, *MSH2*, *MSH6,* and *PMS2* genes or the deletion of the *EPCAM* gene located just upstream of the *MSH2* gene [1–3] are congenitally detected is called Lynch syndrome, and tumors developing in patients with Lynch syndrome are called Lynch-associated tumors (refer to “Lynch syndrome” [4, 5]). On the other hand, sporadic dMMR tumors are mainly caused by acquired hypermethylation in the promoter region of the *MLH1* gene [6].

### Frequencies of dMMR solid tumors by type

Deficient DNA mismatch repair solid tumors can be found in various organs and their frequencies vary widely depending on race, cancer type, disease stage, and whether they are hereditary or sporadic. The frequencies of dMMR solid tumors that were determined by MSI testing or IHC (for testing methods, refer to “dMMR testing methods”) showed large variations among reports, in which the populations analyzed and the testing methods used also differ. In particular, the actual conditions of solid tumors with a low dMMR frequency are not known.

In a report that analyzed 12,019 patients with 32 different types of solid tumors using NGS (for testing methods, refer to “dMMR testing methods”), among the 11 most frequent cancer types, MSI-H tumors accounted for approximately 10% of Stage I–III tumors and approximately 5% of Stage IV tumors [7]. The reported frequencies of MSI-H/MSI-indeterminate (MSI-I) and Lynch-associated tumors determined by analyzing 15,045 patients with over 50 different types of solid tumors at Memorial Sloan Kettering Cancer Center (MSKCC) are shown in Table 2 [8].

**Table 2:** Prevalence of Lynch syndrome by cancer type and MSI status [8]

Cancer type	Total	MSI-H/I (%)	%MSI-H/I Lynch
Total count	15,045	326 (2.2%)	53 (16.3%, 0.35%)
Colorectal	826	137 (16.5%)	26 (19.0%, 3.1%)
Endometrial	525	119 (22.7%)	7 (5.9%, 1.3%)
Small bowel	57	17 (29.8%)	2 (11.8%, 3.5%)
Gastric	211	13 (6.1%)	2 (15.4%, 0.9%)
Esophageal	205	16 (7.8%)	0 (0%, 0%)
Bladder/urothelial	551	32 (5.8%)	12 (37.5%, 2.2%)
Adrenal	44	19 (43.1%)	2 (10.5%, 4.5%)
Prostate	1048	54 (5.1%)	3 (5.6%, 0.29%)
Germ cell	368	33 (9.0%)	1 (3.0%, 0.27%)
Soft tissue sarcoma	785	45 (5.7%)	2 (4.4%, 0.25%)
Pancreatic	824	34 (4.1%)	5 (14.7%, 0.61%)
Mesothelioma	165	6 (3.6%)	1 (16.7%, 0.61%)
CNS tumors	923	30 (3.3%)	1 (3.3%, 0.11%)
Ovarian	343	46 (13.4%)	0 (0%, 0%)
Lung	1952	94 (4.8%)	0 (0%, 0%)
Renal cell	458	11 (2.4%)	0 (0%, 0%)
Breast	2371	150 (6.3%)	0 (0%, 0%)
Melanoma	573	25 (4.3%)	1 (4.0%, 0.17%)
Other cancer type^b^	2816	144 (5.1%)	0 (0%, 0%)

*MSI-I* MSI-indeterminate

^b^Other cancer type includes less common tumors, the majority of which were ampullary carcinoma, anal carcinoma, appendiceal carcinoma, osteosarcoma, peripheral nerve sheath tumor, choriocarcinoma, cervical cancer, neuroendocrine tumor, neuroblastoma, thymic tumor, pheochromocytoma, vaginal carcinoma, Wilms tumor, cancer of unknown primary, head and neck cancer, hepatocellular carcinoma, cholangiocarcinoma, chondrosarcoma, Ewing sarcoma, non-Hodgkin lymphoma, leukemia, and retinoblastoma

### Clinicopathological features of dMMR solid tumors

The association between the conditions of microsatellites and prognoses was weak in a study of 18 types of dMMR solid tumors (5930 cancer exomes) [9]. Besides this study, the outcomes of dMMR solid tumors in various cancers have been analyzed. However, the association with prognoses has not been elucidated.

The clinical features of dMMR solid tumors will be described by the type of cancer below.

#### Clinicopathological features of dMMR gastrointestinal cancer

In Europe and the US, 15% of all colorectal cancers are dMMR [10], and in Japan, 6–7% are dMMR [11, 12]. Among Stage IV cancers, the frequency is low and is reported to be 1.9–3.7% in Japan [13, 14]. Approximately 20–30% of dMMR colorectal cancers are associated with Lynch syndrome and approximately 70–80% are sporadic. Both Lynch-associated and sporadic cancers occur commonly in the right-sided colon and most of them are poorly differentiated adenocarcinoma. As for the association with prognoses, it has been reported that the prognoses of Stage II patients are good and the prognoses of patients for whom curative resection is not possible are poor. The *BRAF* V600E mutation is detected in 35–43% of dMMR colorectal cancers [15], but is rare in Lynch-associated colorectal cancers, even though they are dMMR [6]. (Table 3; for details, refer to “Japanese Society for Cancer of the Colon and Rectum (JSCCR) guidelines 2019 for the treatment of colorectal cancer,” “JSCCR Guidelines 2016 for the Clinical Practice of Hereditary Colorectal Cancer”, and “Japanese Society of Medical Oncology Clinical Guidelines: Molecular Testing for Colorectal Cancer Treatment, Third Edition”).

**Table 3:** Clinicopathological features of dMMR colorectal cancer

	Ratio to dMMR colorectal cancer (%)	*BRAF* mutation	Clinicopathological features
Lynch-associated	20–30	Rarely	More common in juvenile Multiple cancer (synchronous and metachronous) Right-sided colon Poorly differentiated adenocarcinoma
Sporadic	70–80	High frequency	More common in elderly female Right-sided colon Poorly differentiated adenocarcinoma

The frequencies of dMMR tumors in all gastric cancers are high, being approximately 20–25% in Europe and the US and approximately 8–19% in Asian countries [16]. It has been reported that dMMR gastric cancer commonly occurs in elderly women; its main type is distal, intestinal-type adenocarcinoma, and lymph node metastasis and *TP53* mutations are rarely seen [17]. It has also been reported that the prognosis of MSI-H gastric cancer is better than that of MSI-L/MSS gastric cancer (HR 0.76) [18].

The frequencies of dMMR solid tumors in all small intestine cancers are relatively high, being 5–45% [19].

There are only a few reports about esophageal cancer, and no specific views on the frequency or prognosis have been established.

#### Clinicopathological features of dMMR hepato-biliary-pancreatic cancer

Among hepato-biliary-pancreatic cancers, the frequency of dMMR tumors is low and there are a limited number of comprehensive reports. In hepatocellular carcinomas, 1–3% are dMMR tumors, which are found not only in advanced cancers but also in early cancers [7]. It has also been reported that they are high-grade and recur in a short period of time [20]. In biliary tract cancers, the frequency of sporadic MSI-H tumors is reported to be 1.3% [21]. They often develop at a young age [21], and are found among both early and advanced cancers [22]. One report showed that MSI-H tumors had better prognosis than MSS tumors [23], while another report showed that there was no difference in prognosis between these two types of tumors [22]. Thus, there are no consistent views.

Although it was reported from Japan that the frequency of dMMR in pancreatic cancers was 13% [24], recent reports from overseas showed the frequency is 0.8–1.3% [25–28]. Therefore, it is assumed to be around 1% currently. There are some reports showing good prognoses [26, 27], and it is said that dMMR tumors readily respond to immune checkpoint inhibitors [27]. There is also a report that the time to recurrence did not differ between patients receiving and not receiving an adjuvant therapy [29], and another report showed that dMMR pancreatic cancers were poorly differentiated and wild-type *KRAS* was frequently expressed in them [24]. However, the significance of these findings has not yet been elucidated. Clinicopathological features of dMMR hepato-biliary-pancreatic cancers are summarized in Table 4.

**Table 4:** Clinicopathological features of dMMR hepato-biliary-pancreatic cancer

	Clinicopathological features
Lynch-associated	Gall bladder cancer: good prognosis Pancreatic cancer: good prognosis
Sporadic	Hepatocellular carcinomas: high-grade malignancy Bile duct cancer: more common in juvenile Pancreatic cancer: good prognosis

#### Clinicopathological features of dMMR gynecological cancer

In gynecological cancers, dMMR is most commonly seen in endometrial cancer. In the general population, the lifetime risk for endometrial cancer is 3%, while in patients with Lynch syndrome, it is 27–71% [30]. In endometrial cancers, the frequency of dMMR is 20–30%. Approximately 5–20% of these patients have pathogenic variants of the *MMR* gene in the germline, while approximately 80–90% of them are sporadic [31, 32]. A comparison of the clinicopathological features of Lynch-associated endometrial cancers and sporadic endometrial cancers is summarized in Table 5. The analysis of 173 patients with endometrial cancers reported that progression-free survival (PFS) and overall survival (OS) in patients with dMMR endometrial cancers tended to be poorer than those in patients with proficient MMR (pMMR) endometrial cancers (PFS: *P* = 0.057; OS: *P* = 0.076), while in patients with Lynch syndrome, there was no association with prognoses (PFS: *P* = 0.357; OS: *P* = 0.141) [33].

**Table 5:** Clinicopathological features of dMMR endometrial cancer

	Clinicopathological features
Lynch-associated	More common in juvenile and isthmus uteri Endometrioid carcinoma is more common, but there are also clear cell carcinoma, serous carcinoma, and sarcoma Carriers of the *MSH6* pathogenic variant are recognized as having a comparatively high risk of endometrial cancer
Sporadic	Low grade (well-differentiated) endometrioid carcinoma is more common

Whereas for ovarian cancer, the lifetime risk in ordinary groups is 1.5%; for Lynch syndrome, it is 3–20% [30, 34, 35]. In a recent report in Japan, it was stated that a pathogenic variant of an MMR gene was recognized in 2.6% of epithelial ovarian cancer cases [36].

The risk of Lynch syndrome occurring differs according to the gene, but carriers of the MSH6 pathogenic variant are recognized as having a comparatively high risk of endometrial cancer [37, 38].

#### Clinicopathological features of dMMR urological cancer

Of urological cancers, dMMR is most commonly seen in renal pelvic/ureteral cancers, and also seen in prostate cancer, germ cell tumor, and bladder cancer. In renal pelvic/ureteral cancers, the frequency of dMMR is 5–11.3% [39]. Deficient DNA mismatch repair renal pelvic/ureteral cancers are histopathologically characterized by an inverted growth pattern and a low stage, while there are no sites of predilection for these cancers [40]. Lynch-associated renal pelvic/ureteral cancers develop at a younger age and are more common in women than general pelvic/ureteral cancers [41]. There is also a report that more than half of Lynch-associated renal pelvic/ureteral cancers are MSS/MSI-L [41]. Besides renal pelvic/ureteral cancers, it has been reported that some prostate cancers, germ cell tumors, and bladder cancers may be Lynch-associated [39]. Clinical features of sporadic dMMR urological cancers are not known. Clinicopathological features of dMMR urological cancer are summarized in Table 6.

**Table 6:** Clinicopathological features of dMMR urological cancer

	Clinicopathological features
Lynch-associated	Urothelial cancer: more common in juvenile female Prostate cancer and germ cell carcinoma are also lynch-associated cancer
Sporadic	Unknown

### dMMR-testing methods

The dMMR-testing methods include MSI testing, the immunohistochemistry (IHC) for MMR proteins (MLH1, MSH2 MSH6, and PMS2), and NGS testing, as shown below.

#### MSI testing

In the MSI testing method, microsatellite regions of DNA obtained from normal and tumor tissues are amplified by the PCR method and the number of repeats of microsatellite sequence is determined and compared. In practice, the lengths of PCR products, which reflect the number of repeats, are compared in electrophoresis. In a method using a classical Bethesda panel, the lengths of five microsatellite markers (BAT25, BAT26, D5S346, D2S123, and D17S250) are compared between tumor and normal tissues. When the lengths are different, MSI is determined to be positive, and positive MSI for two or more markers is determined to be MSI-H and positive MSI for only one marker is determined to be MSI-L (low-frequency MSI). When no positive MSI is observed for any marker, it is determined to be MSS (microsatellite stable). MMR function in a tumor is judged to be deficient (dMMR) for MSI-H tumors and as proficient (pMMR) for MSI-L/MSS tumors. The Bethesda panel contains three dinucleotide repeat markers, which have been reported to be less sensitive and less specific to MSI than mononucleotide repeat markers. In recent years, in dMMR testing, panels consisting of only mononucleotide repeat markers [pentaplex and the MSI test kit (FALCO)] are often used. BAT25 and BAT26, mononucleotide repeat markers used in many panels, are high in both sensitivity and specificity for MSI [42].

**Fig. 1:**
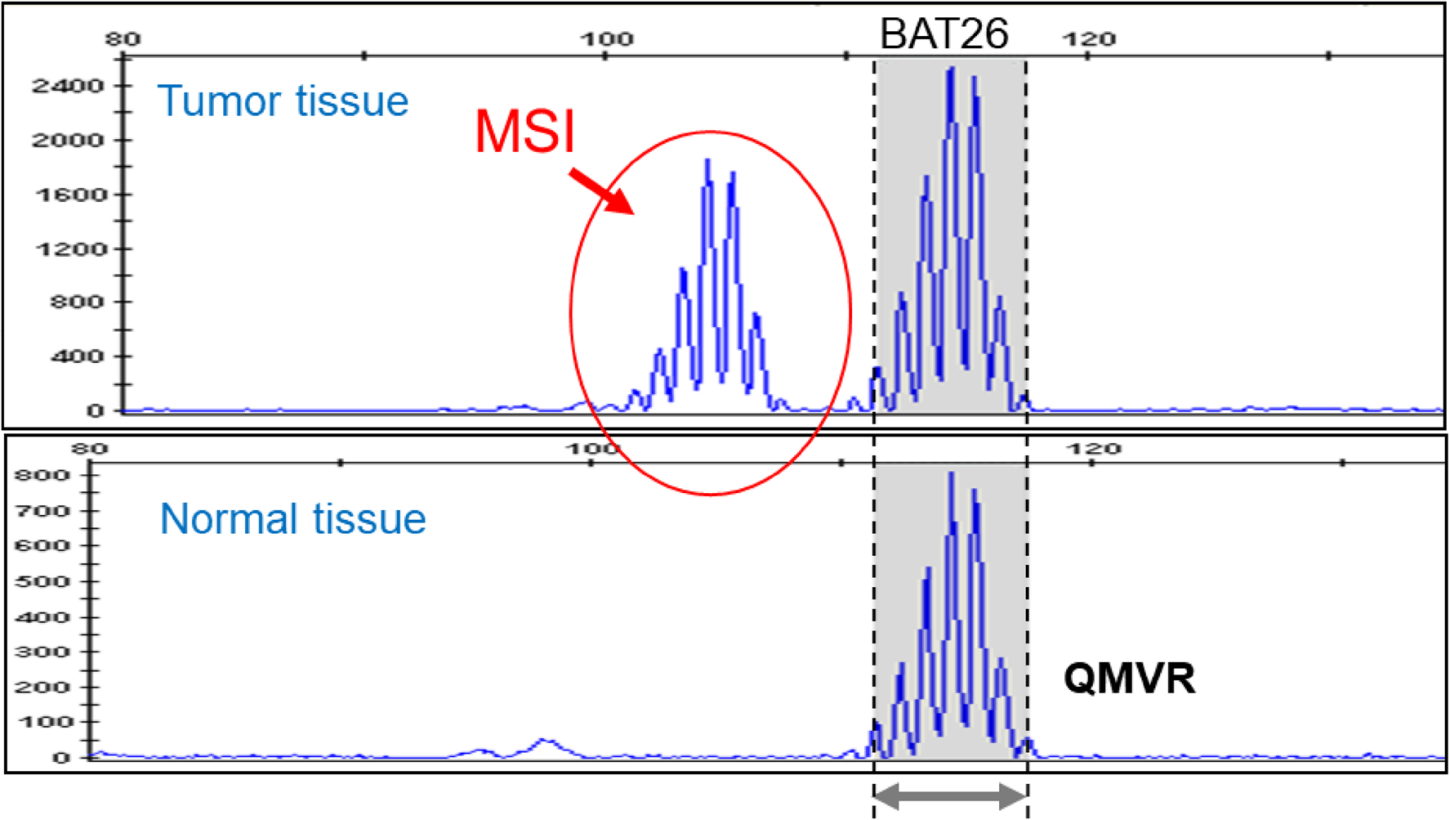
MSI analysis of BAT26. Area with a gray background was QMVR of BAT26. In tumor tissue, the sizes of microsatellites (patterns framed by red lines) are different from those seen in normal tissue

In September 2018, in Japan, “MSI test kit (FALCO)” was approved as a companion diagnostic for pembrolizumab. This test kit adopts a panel consisting of only mononucleotide repeat markers (BAT-25, BAT-26, MONO-27, NR-21, and NR-24) (Table 7). These markers display quasi-monomorphism, and the quasi-monomorphic variation range (QMVR) of each marker is within constant limits irrespective of race (Table 8) [43, 44]. When normal tissues are analyzed with the MSI test kit (FALCO), the length of each microsatellite marker falls within the range of a mean ± 3 bases (QMVR). Therefore, by defining a marker with a length outlying the QMVR as being MSI-positive (Fig. 1), MSI status can be evaluated using only tumor tissues. Actually, for many solid tumors, the MSI-H status determined that only with a tumor tissue was consistent with that determined with a pair of normal and tumor tissues.

**Table 7:** Panel for MSI testing

MSI testing (FALCO)	
Marker	Sequencing structures
BAT25	Mononucleotide repeats
BAT26	Mononucleotide repeats
NR21	Mononucleotide repeats
NR24	Mononucleotide repeats
MONO27	Mononucleotide repeats

**Table 8:** Quasi-monomorphic variation range (QMVR) decided by 149 specimens from healthy Japanese individuals [43]

	NR21	BAT26	BAT25	NR24	MONO27
Japanese	98.4–104.4	111.4–117.4	121.0–127.0	129.5–135.5	149.9–155.9
Patil et al. [44]	98–104	112–118	121–127	129–135	149–155

**Fig. 2:**
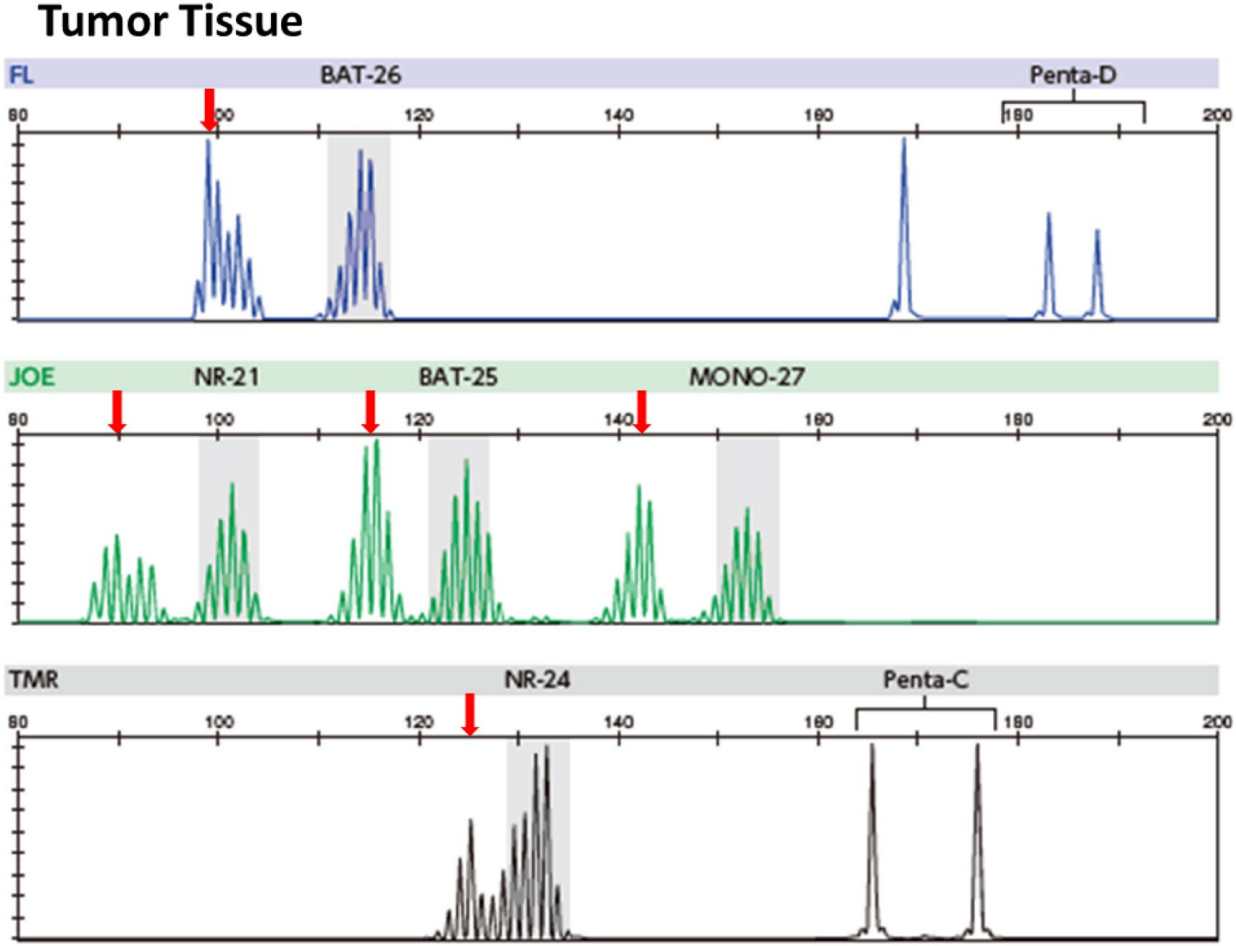
MSI-H case (colorectal cancer). Microsatellite instability (MSI)-positive (↓)

**Fig. 3:**
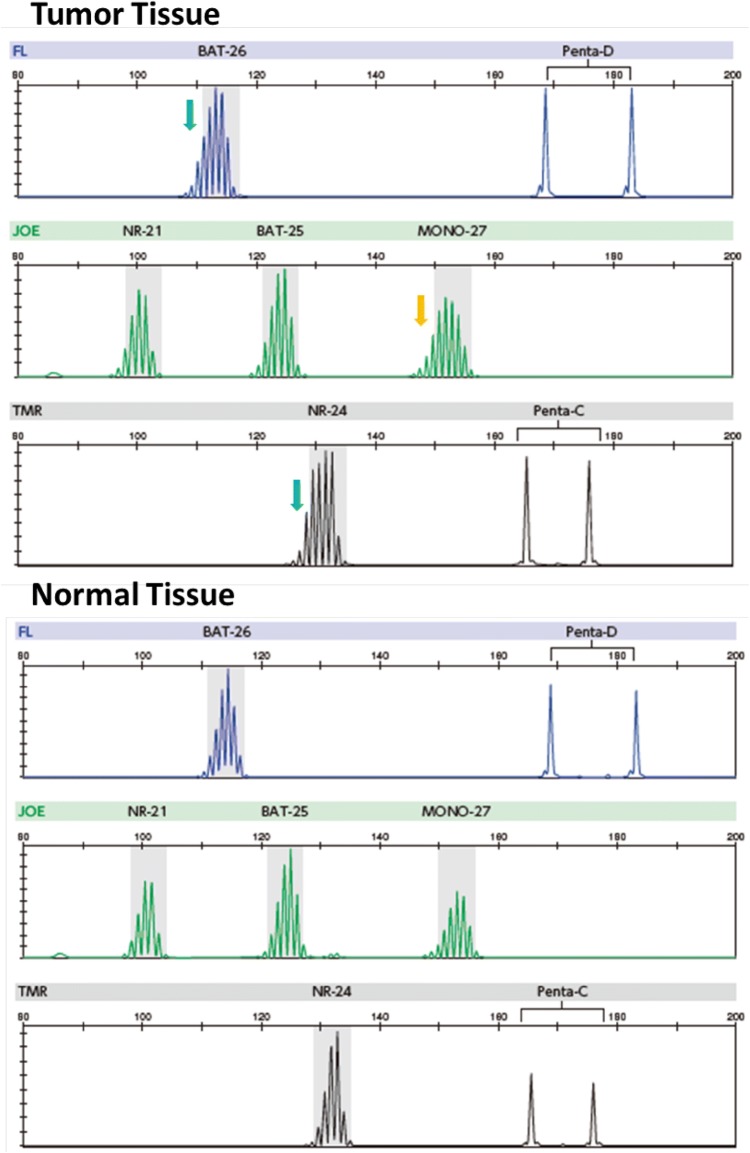
MSI-H case that need attention in decision (endometrial cancer). In tumor tissue, there were two markers (↓) that need attention in decision. In comparison with markers in normal tissue, these patterns were defined as MSI positive. Moreover, there was one additional marker that defined as MSI-positive compared with normal tissue

For colorectal cancer, the concordance rate of the dMMR determination between MSI testing and the IHC for MMR proteins (refer to “Immunohistochemistry for MMR proteins”) has been reported to be ≥ 90%. However, some solid cancers other than colorectal cancer have shown slightly low concordance rates. As a possible cause for this finding, it has been suggested that the extent of altered repeat sequences may vary among organs: on average, a 6-base shift is observed for colorectal cancer (Fig. 2), while only a 3-base shift is observed for other solid tumors (Fig. 3) [45]. The MSI test kit (FALCO) uses the QMVR of the mean ± 3 bases as a criterion for evaluating each marker. Therefore, if the extent of the shift is small, MSI will test false negative. Such false-negative results have been reported for brain tumor, ureteral cancer, uterine body cancer, ovarian cancer, bile duct cancer, and breast cancer. Therefore, MSI testing results need to be interpreted cautiously, particularly when MSI testing is performed with only tumor tissues.

#### Immunohistochemistry (IHC) for MMR proteins

**Fig. 4:**
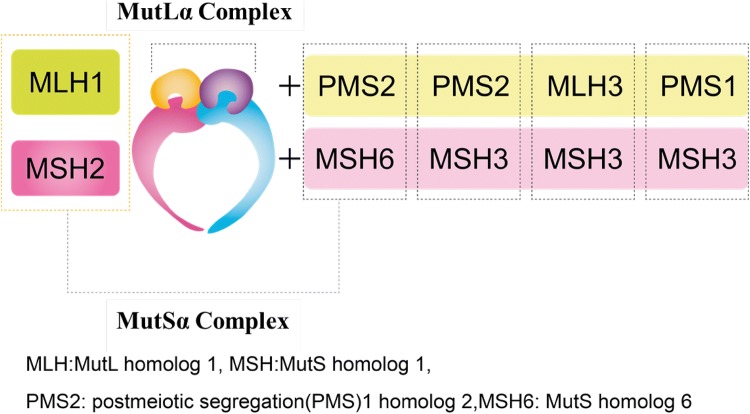
MMR protein human MutLα/MutSα complex

The expression of MMR proteins (MLH1, MSH2, MSH6, and PMS2) in tumor tissue is examined by IHC to evaluate whether the tumor is dMMR. In the evaluation, an internal positive control (the glandular base of the colonic mucosa or the germinal center of a lymphoid follicle in non-tumor tissue) is used to check the appropriateness of staining. If all four proteins are expressed, the tumor is determined to be MMR proficient, and if the expression of at least one protein is lost, the tumor is determined to be dMMR. An advantage of using IHC instead of MSI testing is that genes responsible for dMMR status can be presumed based on the pattern of proteins, whose expression is lost. For example, MSH6 can form a heterodimer only with MSH2. Therefore, if the *MSH2* gene is altered, MSH6 becomes unstable as the protein and becomes degraded, resulting in the loss of both MSH6 and MSH2 expressions in immunohistochemistry. In contrast, MSH2 can form a heterodimer with MSH3, as well as with MSH6. Therefore, even if the *MSH6* gene is altered, MSH2 expression is maintained. Similarly, PMS2 can form a heterodimer only with MLH1, but MLH1 can form heterodimers with proteins other than PMS2 (Fig. 4). In many cases, the staining patterns in Table 9 are displayed. If a staining result does not show any of these patterns, check the appropriateness of staining. If a difficulty arises in judgment, perform additional testing such as MSI testing to make a comprehensive judgment.

**Table 9:** Suspected mutant genes in immunostaining for MMR proteins

	Expression in immunostaining			
	MLH1	MSH2	PMS2	MSH6
Mutant gene				
*MLH1*	−	+	−	+
*MSH2*	+	−	+	−
*PMS2*	+	+	−	+
*MSH6*	+	+	+	−

When staining results other than the patterns in the table are obtained, confirm the adequacy of staining before considering the possibility that the patient is exceptional and perform MSI testing if needed

It is recommended to evaluate four proteins, MLH1, MSH2, MSH6, and PMS2. However, if the evaluation of the four proteins is difficult, because the amount of specimens is limited or for other reasons, screening only with MSH6 and PMS2 is acceptable [46].

#### NGS testing

The evaluation of deficient MMR function using the NGS techniques is broadly divided into methods that target only microsatellite regions and those that evaluate MMR function as part of comprehensive cancer genome profiling. As an example of the former, the MSIplus panel has been reported [47]. This method measures the lengths of a total of 18 different microsatellite marker regions using the NGS technique. If instability is detected in 33% or more of the markers, the condition is judged to be MSI-H.

An example of the latter is the FoundationOne CDx. This method evaluates changes in the lengths of 95 intronic microsatellite markers that were amplified as part of comprehensive cancer genome profiling, to makes a diagnosis. The concordance rate between results from FoundationOne CDx and those from MSI testing or IHC was reported to be 97% [48]. Other methods include the MSIsensor algorithm using MSK-IMPACT [49], the MOSAIC algorithm using whole exome sequencing (WES) [9], and the MANTIS algorithm [50]. These methods determine a condition to be MSI-H differently depending on databases and algorithms regarding the regions to be profiled and the microsatellite markers located in the regions.

#### Specimens suitable for dMMR testing and the number of testing

Recommended specimens are formalin-fixed, paraffin-embedded tissue blocks. If it is histologically confirmed that a sufficient amount of tumor cells for the specific testing method is contained in the relevant tissue, a freshly frozen tissue specimen may be used. There are reports that the concordance rates of determined dMMR status in lymph node metastases were lower than those in liver metastases [51–53], while there are other reports that dMMR-testing results did not differ between primary lesions and metastatic lesions. Based on the mechanisms of tumor development, dMMR is presumed to be present from a relatively early phase. Therefore, the determined dMMR status is considered to be similar between primary lesions and metastatic lesions. When selecting specimens, however, a higher priority should be given to obtaining a sufficient amount of tumor cells than to the methods or sites of specimen collection. For the handling of specimens, refer to “Guidelines on the Handling of Pathological Tissue Samples for Genomic Medicine” and other related documents. Given that MLH1 and MSH6 protein expressions are reported to be lost after treatment with a regimen containing cisplatin [54, 55] when specimens are collected at different timepoints, it is desirable to use specimens that have not yet been modified by pharmacotherapy for dMMR testing.

When multiple primaries, which have more than one primary site, are tested, the determined dMMR status can be different among the primary sites. If cancers are judged to be unresectable and more than one potential primary site is present, more advanced primary sites to be treated earlier should be estimated based on clinical judgement and tested for dMMR. However, if there is more than one primary site candidate, it is desirable to perform a biopsy again on metastatic sites to be treated earlier, to the extent possible, and dMMR testing. In Japan, MSI testing is covered by health insurance when used to screen for Lynch syndrome and to determine the applicability of PD-1/PD-L1 inhibitors. It is also allowed by health insurance to perform MSI testing for one purpose followed by performing MSI testing for another purpose.

### PD-1/PD-L1 inhibitors for dMMR solid tumor

The PD-1 (CD279) molecule, which belongs to the CD28 family, is an immunosuppressive costimulatory signal receptor and was cloned by Honjo et al. [56]. Subsequently, it was found that PD-1 is expressed in activated T cells and B cells and in myeloid cells, inhibits T-cell activity in an antigen-specific manner by binding to its ligand, and plays an important role in peripheral immune tolerance. PD-1 ligands include PD-L1 (CD274 and B7-H1) and PD-L2 (CD273 and B7-DC). The PD-1/PD-L1 pathway is the main immunoregulatory system utilized by cancer cells to escape T-cell immunosurveillance and has been detected in various solid tumors.

**Fig. 5:**
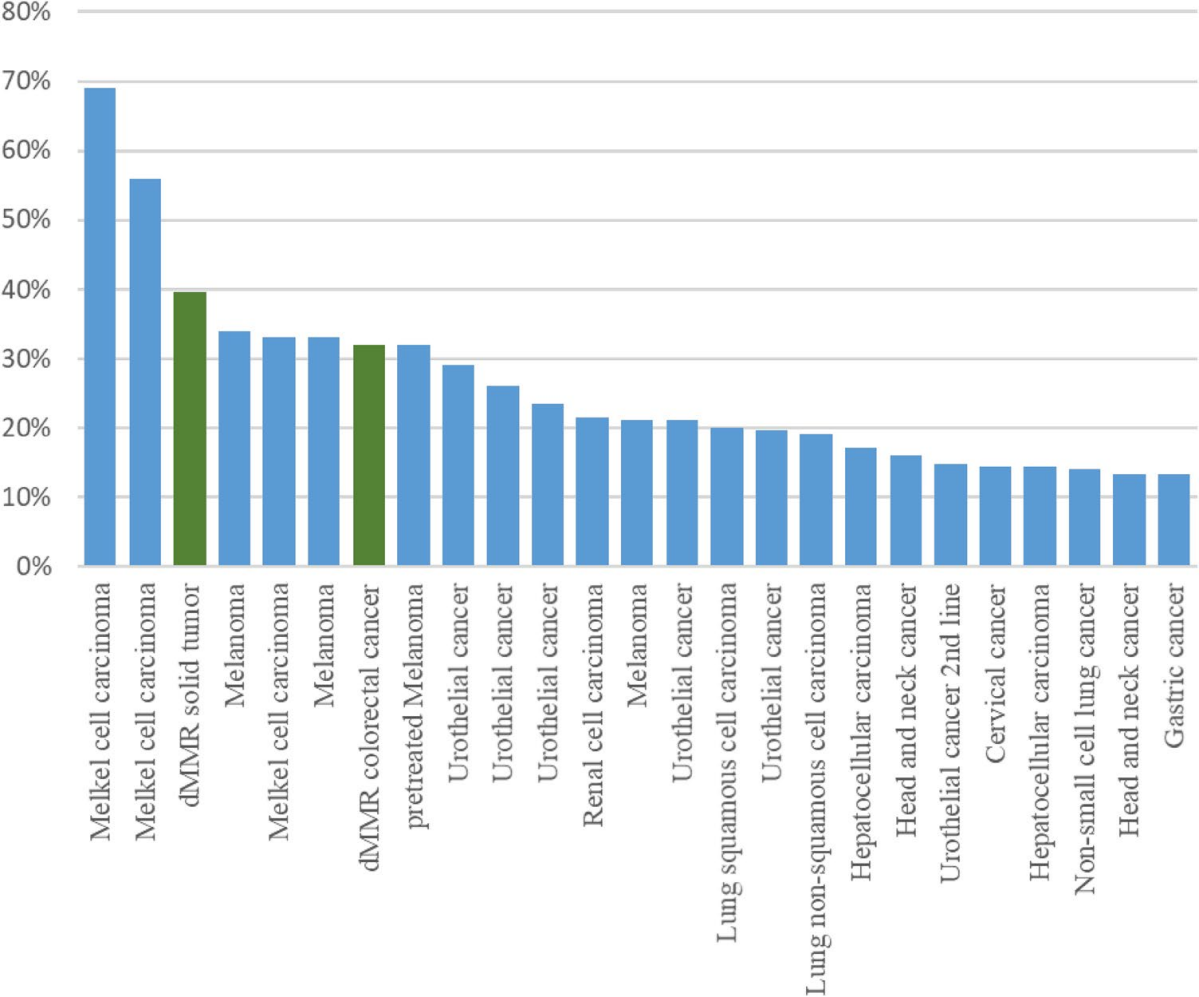
Objective response rate with PD-1/PD-L1 inhibitors by cancer type and trial. Note: each bar represents one clinical trial (green bar: dMMR tumor)

As monoclonal antibody drugs to block this pathway, PD-1 inhibitors (pembrolizumab and nivolumab) and PD-L1 inhibitors (atezolizumab, avelumab, and durvalumab) have been introduced into clinical practice. These drugs exert anti-tumor effects by reactivating anti-tumor immunity through the activation of tumor-specific cytotoxic T lymphocytes (CTL) in the tumor microenvironment. They exert anti-tumor effects through actions different from those of conventional cytotoxic anticancer drugs or molecular targeted drugs. Besides dMMR solid tumors, they were approved for 10 types of solid tumors by FDA and 8 types of solid tumors in Japan as of February 2019 and are used in clinical practice. Previously reported response rates of PD-1/PD-L1 inhibitors for various solid tumors are summarized in Fig. 5.

In dMMR solid tumors, genomic alterations occur with high frequency due to deficient MMR function, which sometimes leads to the synthesis of proteins with altered amino acids, parts of which are presented as antigenic peptides by human leukocyte antigens (HLA). These new antigens, called neoantigens, are recognized as non-self and activate Th1/CTL in tumor tissues. On the other hand, the expression of immune checkpoint molecules including PD-1 is induced, as a negative feedback. Thus, in dMMR solid tumors, regulatory mechanisms against tumors by the immune system play an important role in the suppression. Therefore, PD-1/PD-L1 inhibitors are expected to be effective.

The KEYNOTE-016 study was a phase II study to explore the efficacy and safety of pembrolizumab in patients with all solid tumors including colorectal cancer, and the outcomes from 86 patients with 12 types of dMMR solid tumors have been reported [7]. The outcomes were good with an objective response rate (ORR) of 53% (95% CI 42–64%) and a complete response (CR) of 21%. Neither median progression-free survival (PFS) nor median overall survival (OS) was reached and no obvious differences were detected among different types of solid tumors [7].

Moreover, the KEYNOTE-164, a phase II study of pembrolizumab in patients with dMMR colorectal cancers, was conducted with two cohorts, i.e., patients who had previously received chemotherapy with fluoropyrimidines, oxaliplatin, and irinotecan hydrochloride hydrate (cohort A) and those who had previously received 1 or more regimens of chemotherapy (cohort B). The treatment outcomes of 61 patients in cohort A were good with an ORR of 28% (95% CI 17–41), a median PFS of 2.3 months (95% CI 2.1–8.1), and the median OS not reached. The median duration of response (DoR) was not reached, and 82% of the patients who responded had a DoR of 6 months or longer [57]. Similarly, in the KEYNOTE-158 study, a phase II study of pembrolizumab in the standard systemic treatment-unresponsive/intolerant patients with dMMR advanced solid tumors, the treatment outcomes of 94 patients were good with an ORR of 37% (95% CI 28–48), a median PFS of 5.4 months (95% CI 3.7–10.0), and a median OS of 13.4 months (95% CI ≥ 10.0, upper limit not reached), demonstrating efficacy irrespective of cancer types. Moreover, the median DoR was not reached, and 51% of the patients who responded had a DoR of 6 months or longer, demonstrating the sustained efficacy [58].

Adverse events were observed in 57.4% of the patients in the KEYNOTE-164 study. Common adverse drug reactions (≥ 10%) were arthralgia (16.4%), nausea (14.8%), diarrhea (13.1%), asthenia (11.5%), and pruritus (11.5%) [57]. In the KEYNOTE-158 study, adverse events were observed in 61.7% of the patients, and common adverse drug reactions (≥ 10%) were fatigue (11.7%) and pruritus (11.7%) [58]. Moreover, in a report on the incidences of adverse events at the time of the approval of the additional indication of pembrolizumab for MSI-H solid tumors (including patients with malignant melanoma, non-small cell lung cancer, classical Hodgkin’s lymphoma, and urothelial cancer), adverse events of Grade 3 or higher were observed in 20.7% of the patients, and those observed in ≥ 1% of the patients were neutropenia (2.9%), thrombocytopenia (1.3%), diarrhea (1.4%), pneumonitis (1.4%), and malaise (1.3%). Unlike conventional anticancer drugs, not only adverse events such as arthritis, nausea, malaise, and pruritus, but also unique autoimmune disease-like immune-related adverse events (irAEs) may occur. Therefore, careful whole-body management is required (for details, refer to the “Management of toxicities from immunotherapy: JSMO Clinical Practice Guidelines for diagnosis, treatment and follow-up”).

## Lynch syndrome

Lynch syndrome is an autosomal dominant hereditary disease caused by pathogenic variants of the *MMR* gene in the germline. Lynch syndrome is a rare disease, accounting for 2–4% of all colorectal cancers according to reports from Europe and the US. However, since various malignant tumors including colorectal cancer and endometrial cancer develop in patients and their family (Table 10), it is clinically important to diagnose Lynch syndrome.

**Table 10:** Cumulative lifetime risk of Lynch syndrome-associated neoplasms

Cancer subtype	Cumulative risk (%)
Colorectal cancer	54–74% (male), 30–52% (female)
Endometrial cancer	28–60%
Gastric cancer	5.8–13%
Ovarian cancer	6.1–13.5%
Small-bowel cancer	2.5–4.3%
Bile duct cancer	1.4–2.0%
Pancreatic cancer	0.4–3.7%
Urothelial cancer	3.2–8.4%
Brain tumor	2.1–3.7%
Sebaceous gland tumor	1–9%^a^

^a^Partial Amendment of JSCCR guidelines 2016 for the clinical practice of hereditary colorectal cancer

In patients with Lynch syndrome, one allele of the MMR gene has a pathogenic variant of the germline. If the other wild-type allele acquires a loss-of-function alteration (including methylation in the promoter region), MMR function is lost, this is considered to contribute to cancerization.

In Japan, if clinical information of a patient meets the Amsterdam Criteria II (Supplemental Table S1) or the revised Bethesda Guidelines (Supplemental Table S2), MSI testing or IHC, are recommended for the secondary screening (Supplemental Fig. S1). In Europe and the US, a universal screening in which MSI testing or IHC is performed in all (or ≤ 70 years) patients with colorectal cancer or endometrial cancer, irrespective of the presence of findings suggesting Lynch syndrome has been proposed.

If the result of MSI testing or IHC suggests Lynch syndrome, the genetic testing of the MMR gene should be considered for definitive diagnosis. If genetic testing is conducted, it is recommended to properly select subjects to be tested (the patient and relatives) and to provide them with genetic counseling before and after genetic testing. Some patients have genetic alterations that are not detectable by the current genetic testing methods, and a definitive diagnosis of Lynch syndrome cannot be made in these patients. Therefore, results should be interpreted carefully.

[Note: Usefulness of *BRAF* testing in patients who were determined to have dMMR by dMMR testing]

The main reason for sporadic colorectal cancers to become dMMR is an acquired abnormal methylation in the promoter region of the *MLH1* gene. In these cancers, the loss of MLH1/PMS2 protein expression is detected by immunohistochemistry. In 35–43% of MSI-H colorectal cancers, the *BRAF* V600E mutation is detected [15], while in colorectal cancers in patients with Lynch syndrome, almost no *BRAF* V600E mutations are detected even in MSI-H cancers [9]. Therefore, in the medical care for colorectal cancer, if the dMMR-testing result shows MSI-H or the loss of MLH1/PMS2 expression, checking for the *BRAF* V600E mutation helps distinguish Lynch-associated colorectal cancers from sporadic ones [59]. However, caution is needed, because it has been reported that the *BRAF* V600E mutation was detected in some colorectal cancers that developed in patients with Lynch syndrome attributable to the *PMS2* gene. For solid tumors other than colorectal cancer, the usefulness of a differential diagnosis with *BRAF* V600E mutation has not been reported.

## Clinical questions (CQs)

The following requirements have been prepared regarding the dMMR testing performed to select patients who are likely to benefit from PD-1/PD-L1 inhibitors and the administration of them. They are shown in the form of answers to the 11 requirements we formulated followed by their recommendation levels (Table 11).

**Table 11:** Summary of recommendations

Recommendations	Level
1. Patients for whom dMMR testing is recommended	
1-1. For patients with advanced solid tumors who are receiving standard systemic treatment or who have difficulty receiving any standard treatment, dMMR testing is highly recommended to determine eligibility for PD-1/PD-L1 inhibitors	SR
1-2. For patients with unresectable solid tumors, irrespective of MMR status, for which clinical application of PD-1/PD-L1 inhibitors has already been approved, dMMR testing should be considered to determine eligibility for PD-1/PD-L1 inhibitors	ECO
1-3. For patients with solid tumors that are curable with local treatment, dMMR testing for determining eligibility for PD-1/PD-L1 inhibitors is not recommended	NR
1-4. For patients with solid tumors who have already undergone treatment with PD-1/PD-L1 inhibitors, dMMR testing for redetermining eligibility for PD-1/PD-L1 inhibitors is not recommended	NR
1-5. When a tumor is detected in patients already diagnosed with Lynch syndrome, dMMR testing for determining eligibility for PD-1/PD-L1 inhibitors is recommended	R
2. dMMR-testing methods	
2-1. As dMMR testing for determining eligibility for PD-1/PD-L1 inhibitors, MSI testing is highly recommended	SR
2-2. As dMMR testing for determining eligibility for PD-1/PD-L1 inhibitors, IHC is recommended	R
2-3. As dMMR testing for determining eligibility for PD-1/PD-L1 inhibitors, an NGS testing approach for which analytical validity has been established is recommended	R
3. Medical care system	
3-1. It is highly recommended that dMMR testing be conducted in an environment that can ensure technical accuracy and the quality of the results	SR
3-2. It is highly recommended that dMMR testing be conducted in an environment with established genetic diagnostic and genetic counseling systems	SR
3-3. It is highly recommended that immune checkpoint inhibitors are used in an environment, where adequate measures can be taken in response to irAEs	SR

*SR* strong recommendation, *R* recommendation, *ECO* expert consensus opinion, *NR* no recommendation

### CQ1 Patients for whom dMMR testing is recommended


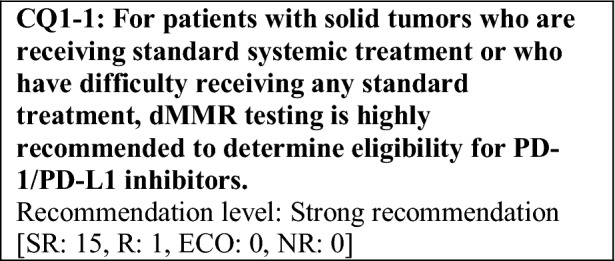


Based on the results of a pooled analysis of 149 patients with advanced/recurrent dMMR solid tumors that progressed after chemotherapy from five clinical studies of pembrolizumab [KEYNOTE-016 study, KEYNOTE-164 study (cohort A), KEYNOTE-012 study, KEYNOTE-028 study, and KEYNOTE-158 study), the United States Food and Drug Administration (FDA) approved pembrolizumab for dMMR solid tumors including colorectal cancers that are resistant to standard systemic treatment or for which no standard treatment is available, on May 23, 2017. In Japan, pembrolizumab was approved on December 21, 2018, based on the updated results of the KEYNOTE-164 study (cohort A) and KEYNOTE-158 study (Table 12).

**Table 12:** Results of the KEYNOTE-164 study (cohort A) and KEYNOTE-158 study [53, 54]

	*N*	Response rate
		*n* (%)
Colorectal cancer	61	17 (28%)^a^
Non-colorectal cancer	94	35 (37%)^b^
Endometrial cancer	24	13 (54%)
Gastric cancer	13	6 (46%)
Small-bowel cancer	13	4 (31%)
Pancreatic cancer	10	1 (10%)
Bile duct cancer	9	2 (22%)
Adrenocortical cancer	3	1 (33%)
Mesothelioma	3	0 (0%)
Small cell lung cancer	3	2 (67%)
Cervical cancer	2	1 (50%)
Neuroendocrine carcinoma	2	0 (0%)
Thyroid cancer	2	0 (0%)
Urothelial cancer	2	1 (50%)
Brain tumor	1	0 (0%)
Ovarian cancer	1	0 (0%)
Prostate cancer	1	0 (0%)
Retroperitoneal tumor	1	1 (100%)
Salivary gland cancer	1	1 (100%)
Sarcoma	1	1 (100%)
Testicular tumor	1	0 (0%)
Tonsil cancer	1	1 (100%)

^a^ORR for dMMR colorectal cancer 95% CI 17–41%

^b^ORR for dMMR non-colorectal cancer 95% CI 28–48%

A study of nivolumab monotherapy and nivolumab/ipilimumab (an anti-CTLA4 antibody drug) combination therapy in patients with dMMR colorectal cancers (the CheckMate-142 study) reported good outcomes with the ORRs of 31% and 55%, respectively, and the median PFSs was not reached in either group [60, 61]. A therapeutic effect was observed irrespective of the degree of PD-L1 expression, the presence of the *BRAF/KRAS* mutations, and the presence of Lynch syndrome. Patient evaluation using EORTC QLQ-C30 demonstrated improved QOL and clinical symptoms [60, 61]. Based on these results, the FDA approved nivolumab monotherapy in August 2017 and nivolumab/ipilimumab combination therapy in July 2018 for metastatic dMMR colorectal cancers that progressed after treatment with fluoropyrimidine, oxaliplatin, and irinotecan. For durvalumab, a PD-L1 inhibitor, a phase II study in patients with dMMR colorectal cancers and phase I/II studies in patients with dMMR solid tumors were conducted and demonstrated an efficacy with the ORR for colorectal cancers of 22% and an overall ORR of 23% [62]. Efficacy for dMMR solid tumors was reproduced in case reports and the analyses of dMMR subgroups in prospective phase II studies.

Because the efficacy of PD-1/PD-L1 inhibitors for dMMR solid tumors was demonstrated in patients who had received chemotherapy, these drugs cannot be treatment options for the first-line treatment. Considering the turnaround time (TAT) of dMMR testing, it is desirable to start first-line treatment (standard systemic treatment) established for each organ without waiting for the result of dMMR testing, in principle. In some organs, however, first-line treatments using molecular targeted drugs are selected based on genetic testing results using tumor tissue specimens, for example, HER2 testing for gastric cancer and *RAS/BRAF* testing for colorectal cancer. In such cases, performing dMMR testing along with these tests is considered to be appropriate in terms of the utilization of limited tumor tissue specimens and not losing a therapeutic opportunity with PD-1/PD-L1 inhibitors in the future. On the other hand, as for non-small cell lung cancer, the amount of tumor tissue specimens available for genetic testing is limited in some cases. In such cases, a search for biomarkers, such as the expression of EGFR, ALK, and PD-L1, which is more important than dMMR testing, has priority.

As for dMMR colorectal cancer, the KEYNOTE-164 study reported good outcomes not only in patients who had received chemotherapy with fluoropyrimidines, oxaliplatin, and irinotecan hydrochloride hydrate (cohort A), but also in 63 patients who had received one or more regimens of chemotherapy (cohort B) with the ORR of 32% (95% CI 21–45), the median PFS of 4.1 months (95% CI ≥ 2.1, upper limit not reached), and the median OS not reached. Therefore, the use of pembrolizumab in second- or later-line treatment is considered. Moreover, a phase III study comparing standard systemic treatment and pembrolizumab therapy in patients receiving first-line treatment is underway. If this study demonstrates the efficacy of pembrolizumab in first-line treatment for dMMR colorectal cancers, dMMR testing before the start of first-line treatment would be desirable.

The efficacy of PD-1/PD-L1 inhibitors has been confirmed consistently in dMMR solid tumors, although these reports did not have a sufficient number of patients by cancer type or by treatment line. Molecular biology also suggests a commonly high immunogenicity in dMMR solid tumors. As for adverse events, although caution is needed for the serious immune-related adverse events that often occur, they are generally tolerable. Therefore, for all patients with dMMR solid tumors, including tumors for which PD-1/PD-L1 inhibitors have no approved organ-specific indications from the viewpoint of efficacy and safety, PD-1/PD-L1 inhibitors can be a potent treatment option. The previous clinical studies were conducted in patients who had difficulty receiving standard systemic treatment (including patients with treatment resistance, intolerance due to adverse events, and not treated at patients’ request). When cancer progresses, the patient’s general condition is often worsened. Considering the TAT of dMMR testing, it is desirable to perform dMMR testing early to determine eligibility for PD-1/PD-L1 inhibitors.

Based on the above considerations, for patients with solid tumors who are receiving standard systemic treatment or who have difficulty receiving any standard treatment, dMMR testing is highly recommended to determine eligibility for PD-1/PD-L1 inhibitors.


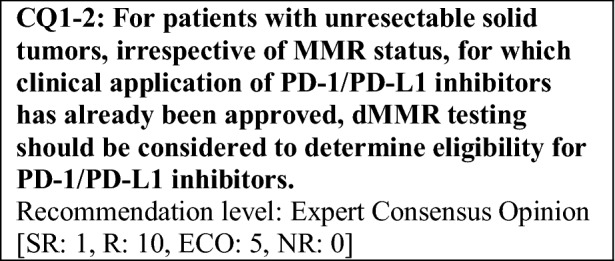


As of April 2019, Table 13 shows the types of solid tumors for which PD-1/PD-L1 inhibitors can be used in clinical practice or are expected to be used in the future (as of April 2019).

**Table 13:** Cancer type for which PD-1/PD-L1 inhibitors can be used in clinical practice (in brackets are field application procedure for approval (as of April 2019)

Cancer type	Biomarker	Treatment line	Agent
Melanoma	None	1st line	Nivolumab Pembrolizumab
Non-small cell lung cancer	PD-L1 positive^a^	1st line	Atezolizumab Durvalumab Nivolumab Pembrolizumab
Renal cell carcinoma	None	2nd line	Nivolumab
		1st line	(Avelumab) (Pembrolizumab)
Head and neck cancer	None	2nd line	Nivolumab Pembrolizumab
Gastric cancer	None	3rd line	Nivolumab
Mesothelioma	None	2nd line	Nivolumab
Urothelial cancer	None	2nd line	Pembrolizumab
Merkel cell carcinoma	None	1st line	Avelumab
Small cell lung cancer	None	1st line	(Atezolizumab)
Breast cancer	PD-L1 positive	1st line	(Atezolizumab)

^a^When using alone as 1st line treatment

For solid tumors for which PD-1/PD-L1 inhibitors can be used in second- or later-line treatment irrespective of MMR function, the applicability of PD-1/PD-L1 inhibitors is judged irrespective of MMR function. Therefore, in principle, it is not necessary to perform dMMR testing. For gastric cancer, nivolumab therapy is recommended in third- or later-line treatment irrespective of the presence of microsatellite instability, but only for dMMR cancer, the guidelines recommend the use of the therapy in second- or later-line treatment [63]. Thus, if the treatment line of PD-1/PD-L1 inhibitors is expected to become earlier depending on MMR function, administration of dMMR testing is also considered.

**Fig. 6:**
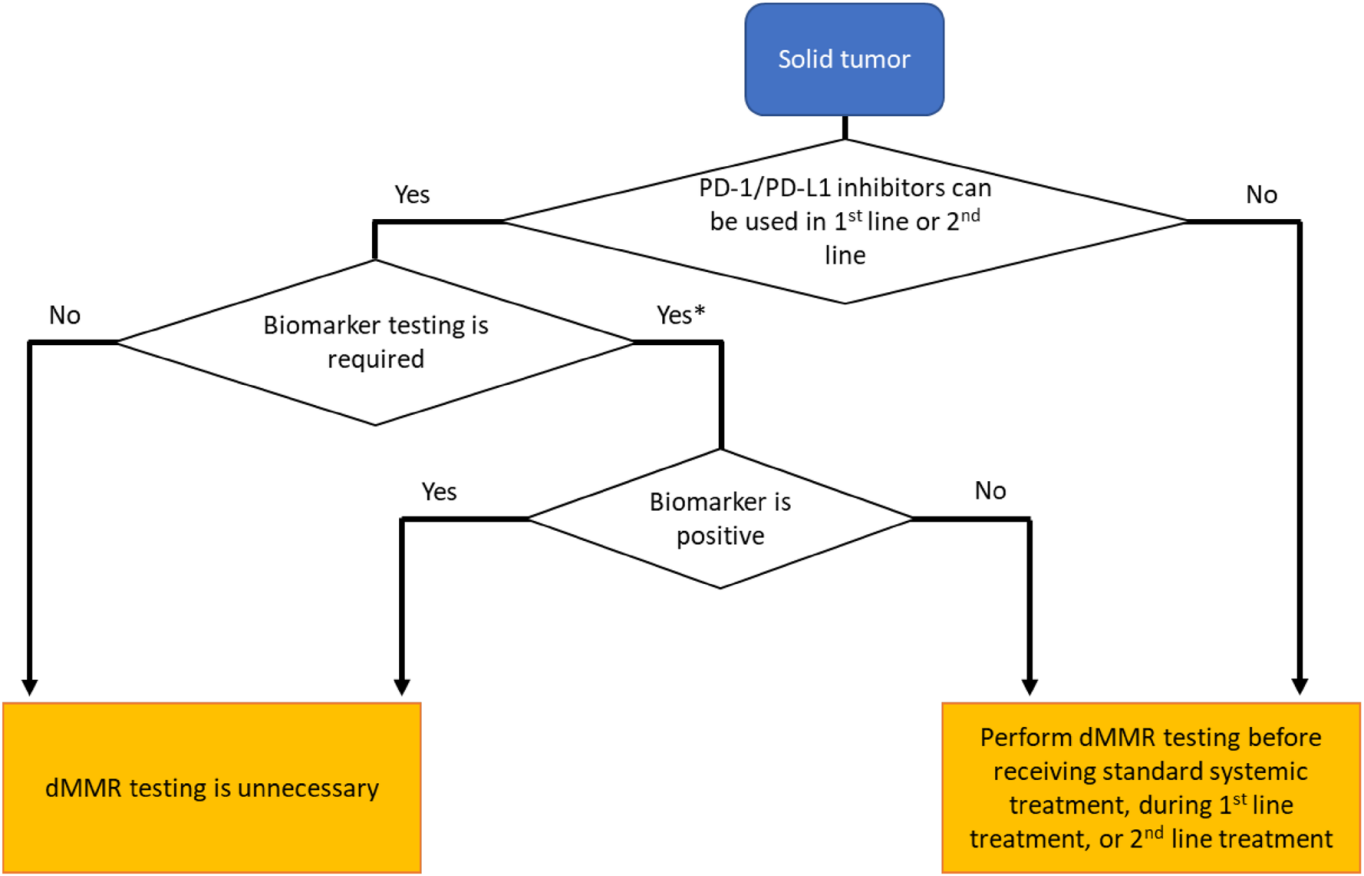
Recommendations by cancer type. *Since biomarkers, such as expression of PD-L1, have different priorities, you should note to perform biomarker testing and dMMR testing at the same time or sequentially


If there is a solid tumor for which the applicability of PD-1/PD-L1 inhibitors is judged based on a biomarker other than the dMMR status such as PD-L1 expression and the biomarker is negative, dMMR testing is recommended, because PD-1/PD-L1 inhibitors are expected to be effective if the tumor is dMMR, as shown in Fig. 6.

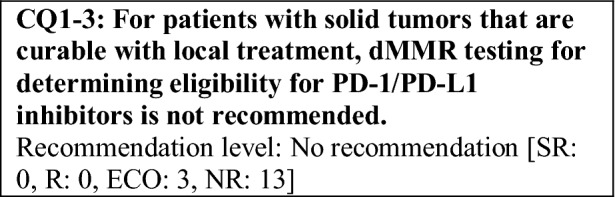


For malignant melanoma, PD-1 inhibitors have demonstrated efficacy as adjuvant therapy and have been approved (KEYNOTE-054 study [64] and ONO-4538-21 study [65]). For non-small cell lung cancer, durvalumab, a PD-L1 inhibitor, has been approved based on the results of the PACIFIC study, a randomized, double-blind, placebo-controlled, multicenter phase III study of durvalumab administered sequentially in patients with unresectable locally advanced cancer (stage III) who did not show disease progression after curative concurrent chemoradiotherapy (CRT) using platinum drugs [66]. However, since no difference in efficacy due to MMR function has been reported from these studies, dMMR testing before treatment is not necessary in principle. For other solid tumors, the efficacy of immune checkpoint inhibitors as perioperative treatment has not been established. Therefore, if the tumor is curable with local therapy, dMMR testing to select therapeutic drugs is not necessary in principle. Thus, at present, for patients with solid tumors that are not locally advanced or metastatic, dMMR testing for determining eligibility for PD-1/PD-L1 inhibitors is not recommended.

However, it is known that dMMR is a favorable prognostic factor for colorectal cancer, particularly for stage II colon cancer, and if the cancer is dMMR, adjuvant therapy with fluoropyrimidines is unnecessary. Therefore, it is considered to be desirable to perform dMMR testing to judge the necessity of adjuvant chemotherapy (for details, refer to “Guidance on Genetic Testing in the Clinical Practice of Colorectal Cancer, Third Edition”). Moreover, currently, a study to verify the efficacy of perioperative use of immune checkpoint inhibitors and a study to concurrently use immune checkpoint inhibitors and chemoradiotherapy for locally advanced cancer are underway. If good outcomes are obtained from these studies, dMMR testing will be necessary for solid tumors curable with local therapy.


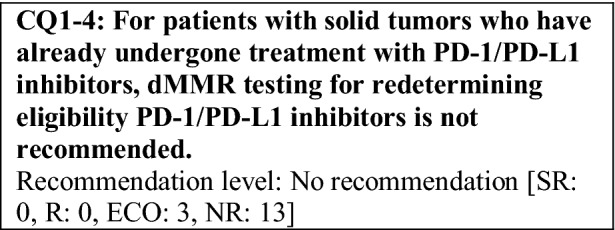


For some solid tumors, PD-1/PD-L1 inhibitors have been approved irrespective of MMR function. The effectiveness of a PD-1/PD-L1 inhibitor in patients who have already received another PD-1/PD-L1 inhibitor has not been demonstrated. Therefore, dMMR testing for the purpose of administration of PD-1/PD-L1 inhibitors in patients with solid tumors who have already received a PD-1/PD-L1 inhibitor is not recommended.


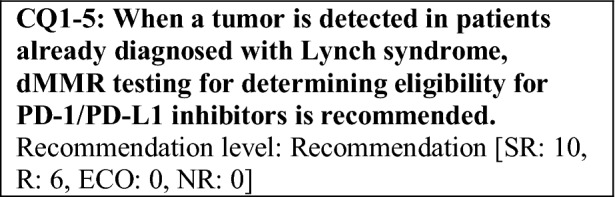


Although the frequency of dMMR is high (80–90%) in Lynch-associated colorectal cancers [67], not all tumors that develop in patients with Lynch syndrome have dMMR. Because the efficacy of PD-1/PD-L1 inhibitors is influenced by the MMR function of the tumor, PD-1/PD-L1 inhibitors are not expected to be effective for pMMR tumors even in patients with Lynch syndrome. Therefore, dMMR testing for determining eligibility for PD-1/PD-L1 inhibitors is also recommended for tumors that develop in patients with Lynch syndrome.

### CQ2 dMMR-testing methods


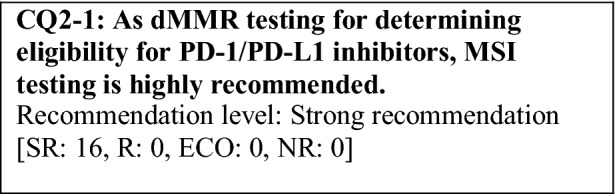


The pooled analysis of patients with dMMR from five KEYNOTE studies (KEYNOTE-016 study, KEYNOTE-164 study (cohort A), KEYNOTE-012 study, KEYNOTE-028 study, and KEYNOTE-158 study) that enrolled patients who were determined to be dMMR based on IHC or MSI testing performed at each study site demonstrated good anti-tumor effect of pembrolizumab. Among 149 patients, 60 patients were determined to be dMMR by MSI testing alone, 47 patients by IHC alone, and 42 patients by both tests [68]. Among them, only 14 patients were determined to be MSI-H by MSI testing performed at a central testing laboratory. A phase II study of nivolumab in patients with colorectal cancer who were determined to be dMMR (the CheckMate-142 study) enrolled patients who were determined to be dMMR by IHC or MSI testing performed at each study site and has demonstrated the efficacy of nivolumab [60]. Thus, if a cancer is determined to be dMMR by either IHC or MSI testing, it is eligible for PD-1/PD-L1 inhibitors, although there may be some differences depending on the type of cancer.

In Japan, in September 2018, “MSI test kit (FALCO)” was approved as a companion diagnostic for pembrolizumab. Any institution in Japan can order this test, and the test is performed in quality-assured testing facilities. Moreover, this test kit can determine the dMMR status by testing tumor tissue alone if tumor cells account for ≥ 40% of the tumor tissue, which is, therefore, very convenient [45]. Thus, as a dMMR-testing method for determining eligibility for PD-1/PD-L1 inhibitors, MSI testing is highly recommended.


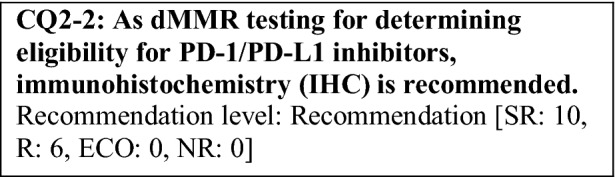


As mentioned above, the efficacy of immune checkpoint inhibitors was demonstrated in patients enrolled in the pooled analysis of five KEYNOTE studies and those in the Checkmate-142 study, who were diagnosed as having dMMR based on IHC or MSI testing performed at each study site. In both analyses, the efficacy of PD-1 inhibitors was demonstrated also in patients who were determined to be dMMR by IHC alone. Actually, in the Checkmate-142 study, in which MSI was determined centrally by MSI testing (with 5 markers used in the Bethesda panel and TGFR type 2), 14 of the 74 patients who were determined to be dMMR at each study site were judged to be non-MSI-H. However, 3 of the 14 patients (21%) responded to treatment [60], and this fact suggests that even when the results of the two tests are not consistent and the dMMR was diagnosed based only on one test, the anti-tumor effect of immune checkpoint inhibitors can be expected. Compared to MSI testing and NGS testing, IHC can be performed inexpensively at individual medical institutions. However, there are some issues. More specifically, as of March 2019, no antibody for IHC has been approved as an in vitro diagnostic in Japan; there are variations in staining depending on the antibodies and staining conditions, and the evaluation method has not been well established. Consequently, IHC is recommended as a dMMR-testing method for determining eligibility for PD-1/PD-L1 inhibitors (however, as of March 2019, no antibody for IHC has been approved as an in vitro diagnostic in Japan).

While a high concordance rate between MSI testing results and IHC results has been reported, some inconsistent cases have been reported. One example is pathogenic missense variants of the MMR genes [69, 70]. In this case, proteins that have lost MMR function are expressed. Therefore, the MSI testing result indicates MSI-H and the tumor is determined to be dMMR, while in IHC, MMR proteins are detected, and the tumor is determined to be MMR proficient (false negative). For this dMMR tumor, PD-1/PD-L1 inhibitors are presumed to be effective. It has been reported that such missense variants are observed in approximately 5% of patients with Lynch syndrome [71]. On the other hand, possible causes of false-negative cases by MSI testing include a low tumor cell ratio. Actually, a tumor cell ratio of ≥ 50% is recommended for the MSI test (FALCO). The positive predictive value of IHC or MSI testing has been reported to be 90.3% [72]. It has been reported that when patients who were diagnosed with dMMR solid tumors by IHC or MSI testing and received PD-1/PD-L1 inhibitors, but did not respond to the therapy were evaluated again by both MSI testing and IHC, 60% of them were found to be MSI-L/MSS/pMMR [72]. To extensively identify patients who can benefit from PD-1/PD-L1 inhibitors, testing should be performed based on a good understanding of the characteristics of both tests. If a false-positive or false-negative result is expected or if there are doubts about the precision or results of the test, performing the other test should be considered.


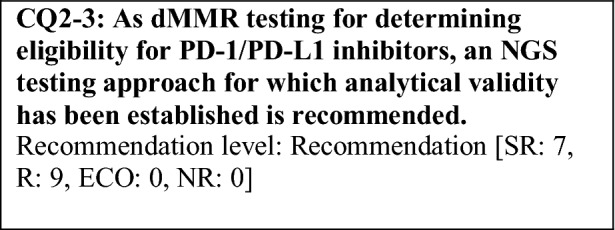


In Japan, on December 27, 2018, the FoundationOne CDx received marketing approval for obtaining comprehensive cancer genome profiles of a tumor tissue from patients with solid tumors and for detecting somatic cell genetic alterations to determine the applicability of some molecular targeted drugs.

Because FoundationOne CDx includes MSI testing using the NGS method, the comprehensive cancer genome profiling and MSI testing (the NGS method) can be performed simultaneously for each cancer type with specimens and at the timing specified in the latest guidelines and other documents issued by relevant academic societies. However, as of March 2019, dMMR testing using the FoundationOne CDx is not covered by health insurance, and there are requirements for facilities to perform the FoundationOne CDx. Therefore, dMMR determination using the NGS method can be accessed at limited facilities in Japan. The FoundationOne CDx also has problems in feasibility. More specifically, it has a certain level of failure rate and needs a large amount of DNA for analysis.

In the five KEYNOTE studies and the Checkmate-142 study conducted for the application for the FDA approval of pembrolizumab, screening tests for dMMR did not include NGS testing. However, the determination of MMR function using NGS testing and MSI testing has a similar measurement principle in that a repeat number of microsatellites is used to determine whether a tumor is dMMR, and it has been reported that the concordance rates between these tests were extremely high, 99.4% in colorectal cancers, and 96.5% in solid tumors other than colorectal cancers [73]. Moreover, when inconsistent cases were analyzed, they were dMMR by IHC, suggesting that NGS testing is more useful. Therefore, it is scientifically unnecessary to perform testing using the MSI test kit (FALCO), a companion diagnostic, or IHC to reconfirm the status determined to be MSI-H by NGS testing, for which analytical validity has been established in the determination of MSI. Thus, an NGS testing approach for which analytical validity has been established is recommended as a dMMR-testing method for determining eligibility for PD-1/PD-L1 inhibitors.

[Note: Liquid biopsy test]

The usefulness of liquid biopsy, which uses body fluid samples such as blood and urine to diagnose the condition of a tumor instead of directly using tumor tissues, has also been reported. The blood usually has a certain amount of free DNA, but the amount of free DNA increases in cancer patients. DNA present in plasma, regardless of whether it is from normal cells or tumor cells, is called cell-free DNA (cfDNA). Because cfDNA in a cancer patient contains DNA from tumors, it is often called circulating tumor DNA (ctDNA). Studies that verified tumor tissues and ctDNA using the MSI test kit and NGS testing reported high sensitivity (86–100%) and specificity (99–100%) [74, 75]. If no tumor tissue is available for testing, therefore, a test using ctDNA is expected to detect genetic alterations in tumor cells in a minimally invasive manner and in real time.

[Note: Relationship between TMB/PD-L1 and MMR]

As biomarkers for the efficacy of PD-1/PD-L1 inhibitors, MSI-H, tumor mutation burden-high (TMB-H), and PD-1/PD-L1 protein expression have been reported.

The proportion of these factors (biomarkers) varies among different cancer types and one factor can confound other factors. In a report of the study that verified the associations among MSI (by NGS), TMB, and PD-L1 protein expression in 11,348 patients with solid tumors, the frequency of the factors and how the factors confound each other vary depending on cancer types (Table 14) [73, 76]. At present, the descriptions of related biomarkers in the indications of PD-1/PD-L1 inhibitors are only as follows: “pembrolizumab for advanced/recurrent non-small cell lung cancers [It may be use monotherapy if tumor tests positive for PD-L1. In regard to the PD-L1 expression ratio of tumor cells (Tumor Proportion Score; TPS), become familiar with the “related clinical trials”. It should be tested by pathologists with sufficient experience, in examination facilities, and using vitro diagnostic development.],” and “pembrolizumab for advanced/recurrent MSI-H solid tumors that progressed following cancer chemotherapy.” However, it is very likely that indications based on each biomarker will increase as clinical studies progress and new findings are obtained in the future. Because there was no correlation between the presence of PD-L1 expression and the therapeutic effect of nivolumab in patients diagnosed with dMMR in the Checkmate-142 study [60], PD-1/PD-L1 inhibitors are expected to be effective even when the tumor is negative for PD-L1 expression, as long as it is dMMR.

**Table 14:** Relationships and percentages of concordance among TMB-H, MSI-H, and PD-L1 expression by tumor cells in different cancers [76]

%	TMB-H and MSI-H and PD-L1+ (%)	TMB-H and/or MSI-H and PD-L1+ (%)	TMB-H and PD-L1+ (%)	MSI-H and PD-L1+ (%)	TMB-H and MSI-H (%)
Total	2.9	11.9	11.4	3.4	10.0
Colorectal cancer	12.8	14.6	14.0	13.4	44.2
Oesophago-gastric adenocarcinoma	14.6	16.8	16.8	14.6	27.7
Melanoma	0.0	32.0	32.0	0.0	0.0
Non-small cell lung cancer	0.5	12.7	12.5	0.7	0.8
Endometrial cancer	5.2	10.5	7.6	8.3	31.0

Thus, at present, TMB or PD-1/PD-L1 testing is not essential to determine the applicability of PD-1/PD-L1 inhibitors. However, it is very likely that they will be recommended in the future to further select patients for whom PD-1/PD-L1 inhibitors are expected to be effective.

### CQ3 medical care system


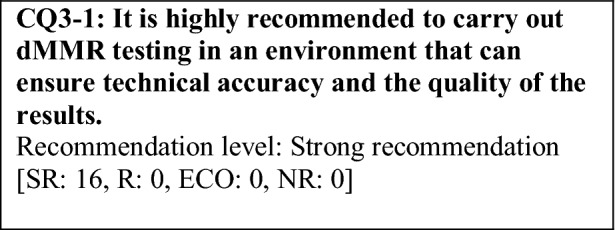


Requirements for the quality assurance of testing need to be considered in terms of facility certification, test details, levels and qualifications of testers, staff education, and risk management. It is desirable that testing facilities ensure the reliability of the precision of testing by obtaining and maintaining ISO 15189 (medical laboratories—requirements for quality and competence), an international standard, or external certifications by the College of American Pathologists (CAP) or other organizations. The quality assurance of test details and testers should be implemented according to the “OECD Guidelines for Quality Assurance in Molecular Genetic Testing,” “Japanese Best Practice Guidelines for Genetic Testing, Commentated Edition,” or other relevant documents. For the handling of specimens, please refer to the “Guidelines on the Handling of Pathological Tissue Samples for Genomic Medicine.”


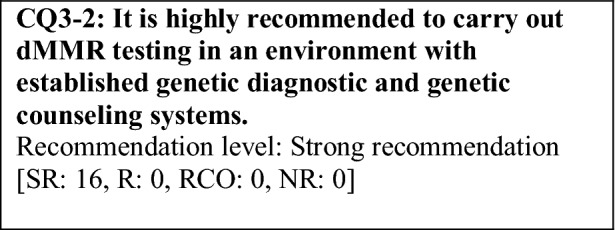


Deficient DNA mismatch repair testing, which is used for determining eligibility for PD-1/PD-L1 inhibitors, has been utilized for screening or as an auxiliary diagnostic method for Lynch syndrome. Therefore, when dMMR testing is performed, informed consent should be obtained after explaining that this test can also be used as screening for Lynch syndrome (refer to “Clinical Practice Resources of the Japanese Society for Familial Tumors” and “Guidance on Genetic Testing in the Clinical Practice of Colorectal Cancer, Third Edition, edited by the Japanese Society of Medical Oncology, November 2016”). As part of the basic clinical practice of cancer, it is assumed that a patient’s family history is taken at the first visit. However, if the patient has been found to have dMMR, the possibility of Lynch syndrome should be reevaluated by checking his or her family history again or through other methods. On the assumption that genetic testing may be considered, a system to provide expert consultation and genetic counseling about the interpretation of test results, subsequent healthcare, heredity in relatives, and other relevant topics must be established in the institution or partner institutions.

Please refer to the following e-learning sites created as part of the construction of a nationwide unified genetic analysis/diagnostic system and the development of a training program by an expert panel consisting of experts from multiple institutions and multiple occupations in collaboration with related academic societies such as the Japanese Society of Medical Oncology:

- e-Learning site about gene-level information regardless of primary tumor site: e-Precision Medicine Japan (https://www.e-precisionmedicine.com).
- e-Learning site about cancer and heredity, and hereditary tumors: Hereditary Tumors e-Learning (https://www.e-precisionmedicine.com/ja/familial-tumors).

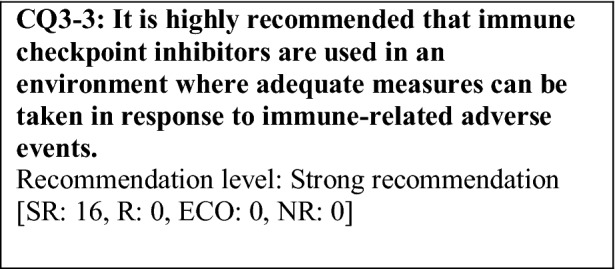


Immune checkpoint inhibitors activate and maintain tumor immunity by blocking co-inhibitory molecules, which work to suppress immunity in various immune cells. Unlike conventional cytotoxic anticancer drugs or molecular targeted drugs, they do not act directly on cancer cells. They exert their effect by activating immune cells. Since irAEs may occur due to the activation of immune cells, whole-body management is required. Because a delay in response and treatment can lead to a fatal course, immune checkpoint inhibitors should be administered in an environment, where adequate measures can be taken (for the handling of each adverse event, refer to the “Guidelines for Cancer Immunotherapy”, and for measures in each cancer type, refer to the “Optimal Use Promotion Guidelines” in addition to the “Guidelines for Cancer Immunotherapy”).

It is recommended to meet the following criteria (excerpted from “Optimal Use Promotion Guidelines”):

(1) About institutions

Designated cancer hospitals and advanced treatment hospitals designated by the Minister of Health, Labour and Welfare, designated cancer hospitals designated by the prefectural governor, and other hospitals that have physicians with sufficient experience in cancer treatment including cancer pharmacotherapy.

(2) About a system to manage pharmaceutical information within the hospital

The hospital has full-time staff engaged in the pharmaceutical information management and an established system to promptly implement the following actions: liaison for receiving information from pharmaceutical companies; management of pharmaceutical information including that on efficacy and safety, and provision of information to physicians; reporting of any adverse events if they should occur; and others.

(3) About the handling of adverse drug reactions

The hospital has an established 24-h clinical care system that can promptly provide proper diagnosis and treatment of adverse drug reactions in case serious adverse drug reactions occur. Because there are a variety of irAEs, a system to cooperate with experts specializing in respective organs and pathologies needs to be established at the institution or partner institutions. Moreover, it is desirable to have an established team medical care system in which healthcare professionals who are engaged in cancer-related clinical practice and have specialized knowledge and skills perform screening for pain, including monitoring for adverse drug reactions, and share the information with attending physicians.

## Conclusion

Many clinical trials have reported the efficacy of immune checkpoint inhibitors in the treatment of dMMR advanced solid tumors. However, there are some issues related to administering immune checkpoint inhibitors in the clinical setting. We have prepared a provisional clinical opinion that proposes the requirements to perform the dMMR testing properly to select patients who are likely to benefit from immune checkpoint inhibitors and to administer them safely.

## Remarks

### Global status of approval of immune checkpoint inhibitors for patients with dMMR solid tumors (as of February 2019)

The approval status in Japan and by the FDA are shown in Supplemental Tables S3 and 4.

### Recommendations in various guidelines

#### The NCCN guidelines (as of February 2019)

Recommendations for tests for individual cancer types, recommendations for PD-1/PD-L1 inhibitors, and whether organ-specific approval has been obtained for PD-1/PD-L1 inhibitors are shown in Supplemental Table S5.

#### ESMO guidelines

##### ESMO consensus guidelines for the management of patients with metastatic colorectal cancer

*Recommendation: MSI testing*

- MSI testing in the metastatic disease setting can assist clinicians in genetic counseling.
- MSI testing has strong predictive value for the use of immune checkpoint inhibitors in the treatment of patients with mCRC.

##### Pan-Asian adapted ESMO consensus guidelines for the management of patients with metastatic colorectal cancer

*Recommendation: Tumour mismatch repair (MMR) testing*

- Immunohistochemistry (IHC) tests for MMR proteins or PCR tests for microsatellite instability (MSI) in the metastatic disease setting can assist clinicians in genetic counseling.
- Tumour MMR testing has strong predictive value for the use of immune checkpoint inhibitors in the treatment of patients with mCRC.

##### ESMO recommendations on microsatellite instability testing for immunotherapy in cancer, and its relationship with PD-1/PD-L1 expression and tumor mutational burden: a systematic review-based approach

Summary of recommendations for MSI testing in the framework of immunotherapy are shown in Supplemental Table S6.

#### Descriptions in guidelines in Japan

Lynch syndrome and screening are described in the “JSCCR guidelines 2019 for the treatment of colorectal cancer,” “JSCCR Guidelines 2016 for the Clinical Practice of Hereditary Colorectal Cancer,” “Japanese Society of Medical Oncology Clinical Guidelines: Molecular Testing for Colorectal Cancer Treatment, Third Edition” and “Guideline for Gynecological Practice in Japan.” (Colorectal cancer-related guidelines also include a description of PD-1 inhibitors.) “The statement for use of pembrolizumab monotherapy in patients with advanced/recurrent MSI-H esophageal or gastric cancer” has been disclosed by Japanese Gastric Cancer Association. “Guidelines for Cancer Immunotherapy” describe immunotherapy, the management of irAEs, and evidence of immunotherapy for individual cancer types (including dMMR solid tumors).

## Electronic supplementary material

Below is the link to the electronic supplementary material.

Supplementary material 1 (DOCX 183 kb)
