# Supplementary material for: Japan Society of Clinical Oncology provisional clinical opinion for the diagnosis and use of immunotherapy in patients with deficient DNA mismatch repair tumors, cooperated by Japanese Society of Medical Oncology, First Edition
Source: Int J Clin Oncol. 2019 Jul 8;25(2):217–39. doi: 10.1007/s10147-019-01498-8 (PMC6989445; doi:10.1007/s10147-019-01498-8)

**Supplementary Table S1. Amsterdam criteria II (1999)**


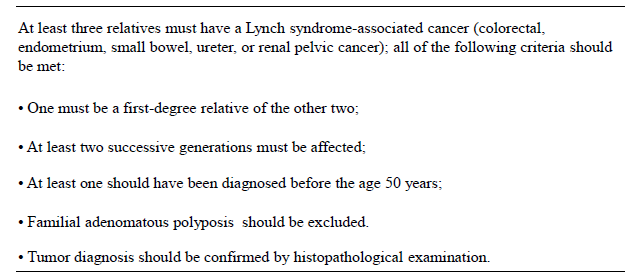


**Supplementary Table S2. The revised Bethesda Guideline (2004)**


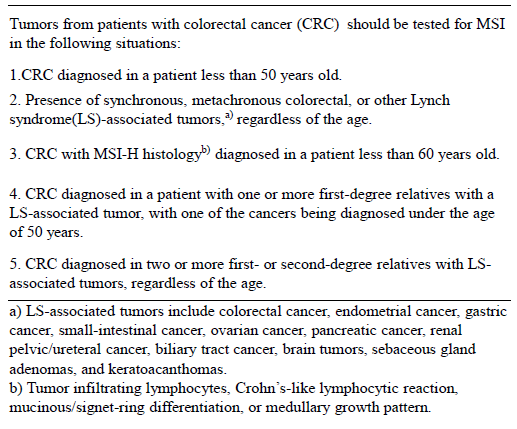


**Supplementary Table S3. Approval status in Japan**

| **Drug** | **INDICATIONS AND USAGE** |
| --- | --- |
| **Pembrolizumab (**KEYTRUDA®**)** | **INDICATIONS AND USAGE**  KEYTRUDA is indicated for the treatment of adult patients with advanced or recurrent MSI-H solid tumors that have progressed following chemotherapy, if refractory or intolerant to standard therapies.  **Note**  1. KEYTRUDA should be administered to patients with advanced or recurrent MSI-H solid tumors based on the result of a companion diagnostic, FALCO MSI testing. Moreover, the test should be carried out in an environment that can ensure technical accuracy and the quality of the results.  2. The safety and effectiveness of KEYTRUDA in patients with MSI-H colorectal cancers that have not progressed following treatment with a fluoropyrimidine, oxaliplatin, and irinotecan have not been established.  3. The safety and effectiveness of KEYTRUDA as first line treatment in patients with MSI-H colorectal cancers have not been established.  4. The safety and effectiveness of KEYTRUDA as adjuvant treatment in patients with MSI-H solid tumors have not been established.  5. The implementation of treatment even aside from the use of this drug should be carefully considered and intended patients should be selected with clear understanding of cancer type that included in clinical trials and the safety and effectiveness of this drug.  **DOSAGE AND ADMINISTRATION**  ・MSI-H Cancer: 200 mg every 3 weeks for adults.  Administer KEYTRUDA as an intravenous infusion over 30 minutes. |

**Supplementary Table S4. Approval status by FDA**

| **Drug** | **INDICATIONS AND USAGE** |
| --- | --- |
| **Pembrolizumab (**KEYTRUDA®**)** | **INDICATIONS AND USAGE**  KEYTRUDA is indicated for the treatment of adult and pediatric patients with unresectable or metastatic, microsatellite instability-high (MSI-H) or mismatch repair deficient  ・solid tumors that have progressed following prior treatment and who have no satisfactory alternative treatment options, or  ・colorectal cancer that has progressed following treatment with a fluoropyrimidine, oxaliplatin, and irinotecan.  This indication is approved under accelerated approval based on tumor response rate and durability of response. Continued approval for this indication may be contingent upon verification and description of clinical benefit in the confirmatory trials.  Limitations of Use: The safety and effectiveness of KEYTRUDA in pediatric patients with MSI-H central nervous system cancers have not been established.  **DOSAGE AND ADMINISTRATION**  ・MSI-H Cancer: 200 mg every 3 weeks for adults and 2 mg/kg (up to 200 mg) every 3 weeks for children.  Administer KEYTRUDA as an intravenous infusion over 30 minutes. |
| **Nivolumab (OPDIVO** **®)** | **INDICATIONS AND USAGE**  ・OPDIVO, as a single agent, is indicated for the treatment of adult and pediatric patients 12 years and older with microsatellite instability-high (MSI-H) or mismatch repair deficient (dMMR) metastatic colorectal cancer (CRC) that has progressed following treatment with a fluoropyrimidine, oxaliplatin, and irinotecan.  ・OPDIVO, in combination with ipilimumab, is indicated for the treatment of adults and pediatric patients 12 years and older with MSI-H or dMMR metastatic CRC that has progressed following treatment with a fluoropyrimidine, oxaliplatin, and irinotecan.  These indications are approved under accelerated approval based on overall response rate and duration of response. Continued approval for these indications may be contingent upon verification and description of clinical benefit in confirmatory trials.  **DOSAGE AND ADMINISTRATION**  ・Microsatellite instability-high (MSI-H) or mismatch repair deficient (dMMR) metastatic colorectal cancer   - OPDIVO 240 mg every 2 weeks.   OPDIVO 3 mg/kg followed by ipilimumab 1 mg/kg on the same day every 3 weeks for 4 doses, then OPDIVO 240 mg every 2 weeks. |
| **Ipilimumab**  **(**YERVOY®**)** | **INDICATIONS AND USAGE**  YERVOY, in combination with nivolumab, is indicated for the treatment of adult and pediatric patients 12 years of age and older with microsatellite instability-high (MSI-H) or mismatch repair deficient (dMMR) metastatic colorectal cancer (CRC) that has progressed following treatment with a fluoropyrimidine, oxaliplatin, and irinotecan. This indication is approved under accelerated approval based on overall response rate and duration of response. Continued approval for this indication may be contingent upon verification and description of clinical benefit in confirmatory trials.  **DOSAGE AND ADMINISTRATION**  ・Microsatellite instability-high (MSI-H) or mismatch repair deficient (dMMR) metastatic colorectal cancer:   - Nivolumab 3 mg/kg followed by YERVOY 1 mg/kg on the same day every 3 weeks for 4 doses, then nivolumab 240 mg every 2 weeks. |

**Supplementary Table S5. Recommendations for dMMR testing and immunotherapy indication for individual cancer type**

| **Guideline** | **Version. Year** | **Testing** | **Immunotherapy Indication** | **Organ-specific approval** | |
| --- | --- | --- | --- | --- | --- |
| **Anal carcinoma** | 2.2018 | MSI/MMR testing is not required. | Subsequent therapy^*+^ | No | |
| **Bladder cancer** | 1.2019 | - | Subsequent therapy* ^+‡†#^ | Yes | |
| **Bone cancer** | 1.2019 | - | Systemic therapy for MSI-H/ MMR-D tumors^*^ | No | |
| **Breast cancer** | 4.2018 | - | - | No | |
| **CNS cancer** | 2.2018 | - | - | No | |
| **Cervical cancer** | 3.2019 | Consider MMR/MSI testing or PD-L1 testing for patients with recurrent, progressive, or metastatic disease. | 2^nd^ line for MSI-H/ MMR-D tumors^*^ | Yes | |
| **Colon cancer** | 4.2018 | Universal MMR or MSI testing is recommended in all patients with a personal history of colon or rectal cancer. | MSI-H/ MMR-D tumors^*+^ | No | |
| **Rectal cancer** | 3.2018 | Universal MMR or MSI testing is recommended in all patients with a personal history of colon or rectal cancer. | MSI-H/ MMR-D tumors^*+^ | No | |
| **Esophageal cancer** | 2.2018 | MMR or MSI testing should be considered on locally advanced, recurrent, or metastatic esophageal adenocarcinoma or EGJ in patients who are candidates for treatment with PD-1 inhibitors. | 2nd line or subsequent therapy for MSI-H/ MMR-D tumors* | No | |
| **Gastric cancer** | 2.2018 | MMR or MSI testing should be considered on locally advanced, recurrent, or metastatic esophageal adenocarcinoma or EGJ in patients who are candidates for treatment with PD-1 inhibitors. | For 2^nd^-line or subsequent therapy for MSI-H/ MMR-D tumors*, for 3^rd^-line or subsequent therapy for PD-L1 positive adenocarcinoma* | Yes | |
| **Head and neck cancer** | 2.2018 | - | 2^nd^-line or subsequent therapy* | Yes | |
| **Hepatobiliary cancer** | 1.2019 | - | MSI-H/ MMR-D tumors^*^  2^nd^-line for patients with HCC who progressed on sorafenib^+^ | Yes, but HCC Only | |
| **Kidney cancer** | 3.2019 | - | 1^st^-line (favorable risk) other recommebdation regimen^+^  1^st^-line (poor/ intermediate risk) preferred regimen^+^ | Yes | |
| **Malignant pleural mesothelioma** | 1.2019 | - | Subsequent therapy^+^ | No | |
| **Melanoma** | 1.2019 | - | 1^st^-line therapy-^*+^ | Yes | |
| **Neuroendocrine and adrenal tumors** | 4.2018 | Adrenocortical carcinoma: Consider MSI or MMR testing | Pembrolizumab should be considered for dMMR or MSI-H unresectable/metastatic adrenocortical tumors that have progressed following prior treatment and have no satisfactory alternative treatment options. | No | |
| **Non-small cell lung cancer** | 3.2019 | - | 1st-line therapy-^*+‡^ | Yes | |
| **Occult primary** | 2.2019 | The population of patients with MSI-H/dMMR occult primary tumors is low. Use IHC for MMR or PCR for MSI, which are different assays measuring the same biological effect. | - | No | |
| **Ovarian cancer** | 2.2018 | - | MSI-H/ MMR-D tumors^*^ | No | |
| **Pancreatic cancer** | 1.2019 | Consider microsatellite instability (MSI) testing and/or mismatch repair (MMR) testing on available tumor tissue | 2nd line for MSI-H/ MMR-D tumors* | No | |
| **Penile cancer** | 1.2019 | - | Subsequent therapy for metastatic/recurrent disease preferred regimen: MSI-H/ MMR-D tumors* | No | |
| **Prostate cancer** | 4.2018 | Consider microsatellite instability (MSI) testing and/or mismatch repair (MMR) testing | Subsequent therapy for MSI-H/ MMR-D tumors* | No | |
| **Small cell lung cancer** | 1.2019 | - | Subsequent therapy*^+^ | Yes | |
| **Soft tissue sarcoma** | 2.2019 | - | Systemic therapy agents and regimens:  Alveolar soft part sarcoma and UPS | No | |
| **Testicular cancer** | 1.2019 | MSI testing if progression after high-dose chemotherapy or 3^rd^-line therapy | Palliative therapy for MSI-H/ MMR-D tumors* | No | |
| **Thymomas and thymic carcinomas** | 1.2019 | - | 2^nd^-line systemic therapy (thymic carcinomas only) | No | |
| **Thyroid carcinoma** | 2.2018 | - | - | No | |
| **Uterine neoplasms** | 3.2019 | For recurrent endometrial cancer, NCCN recommends MSI-H or dMMR testing if not previously done. | Useful in certain circumstances (for MSI-H/ MMR-D tumors)^*^ | No | |
| **Vulvar cancer** | 2.2019 | Consider MMR/MSI testing for patients with recurrent, progressive, or metastatic disease | Chemotherapy for advanced, recurrent/metastatic disease: useful in certain circumstances (2^nd^-line therapy for PD-L1 positive or MSI-H/dMMR tumors)* | No | |
| **Merkel cell carcinoma** | 2.2019 | - | Disseminated disease^*+#^ | Yes |  |

*Pembrolizumab, +Nivolumab, ‡Atezolizumab, †Durvalumab, #Avelumab, - No statement

**Supplementary Table S6. Summary table of recommendations for MSI testing in the framework of immunotherapy and comments**

| **Recommendation A: Immunohistochemistry** | The first test of choice is IHC, using antibodies recognising the four MMR proteins: MLH1, MSH2, MSH6 and PMS2. |
| --- | --- |
| Coefficient of Agreement: strong (8.7) | |
| *Main comment: MMR proteins form heterodimers; for a correct IHC interpretation, the consensus panel highlights that mutations in MLH1 are associated with IHC loss of both MLH1 and PMS2, while mutations in MSH2 are associated with IHC loss of both MSH2 and MSH6. There exist isolated losses of PMS2, MSH2 or MSH6, this strengthening the recommendation to use all four antibodies.* | |
| **Recommendation B: Polymerase Chain Reaction** | In case of doubt of IHC, confirmatory molecular analysis is mandatory. The first-line of molecular analysis is represented by PCR. It can be carried out using two possible panels: (i) a panel with two mononucleotide (BAT-25 and BAT-26) and three dinucleotide (D5S346, D2S123 and D17S250) repeats and (ii) a panel with five poly-A mononucleotide repeats (BAT-25, BAT-26, NR-21, NR-24, NR-27). The five poly-A panel is the recommended panel given its higher sensitivity and specificity. |
| Coefficient of agreement: strong (8.6) | |
| *Main comment: both the suggested panels have been and are being used to assess MSI in clinical trials. Molecular tests guarantee the highest values of specificity and sensitivity in MSI testing.* | |
| **Recommendation C: Next-generation Sequencing** | NGS represents another type of molecular tests to assess MSI. Its main advantages are represented by the possibilities of coupling MSI analysis with the determination of tumour mutational burden (TMB). |
| Coefficient of agreement: very strong (9.0) | |
| *Main comment: NGS should be carried out only in selected centres devoted to these techniques.* | |

Coefficient of agreement ranges from 0 = totally disagree, to 10 = totally agree.

Abbreviations: IHC: immunohistochemistry; PCR: polymerase-chain reaction; NGS: next-generation sequencing.

**Supplementary Figure S1. Diagnostic procedures for Lynch syndrome (JSCCR Guidelines 2016 for the Clinical Practice of Hereditary Colorectal Cancer)**


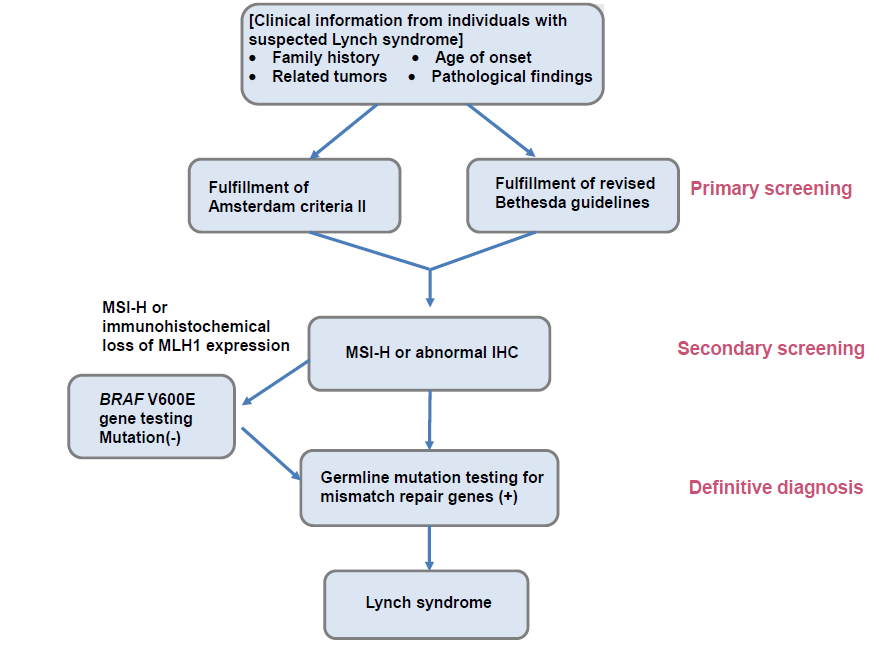

Supplement: Supplementary file 1 — Supplementary material 1 (DOCX 183 kb) [file 10147_2019_1498_MOESM1_ESM.docx]
